# Signal transduction pathway mutations in gastrointestinal (GI) cancers: a systematic review and meta-analysis

**DOI:** 10.1038/s41598-020-73770-1

**Published:** 2020-10-30

**Authors:** Alireza Tabibzadeh, Fahimeh Safarnezhad Tameshkel, Yousef Moradi, Saber Soltani, Maziar Moradi-Lakeh, G. Hossein Ashrafi, Nima Motamed, Farhad Zamani, Seyed Abbas Motevalian, Mahshid Panahi, Maryam Esghaei, Hossein Ajdarkosh, Alireza Mousavi-Jarrahi, Mohammad Hadi Karbalaie Niya

**Affiliations:** 1 Department of Virology, Faculty of Medicine, Iran University of Medical Sciences, Tehran, Iran; 2 Student Research Committee, Iran University of Medical Sciences, Tehran, Iran; 3 Gastrointestinal and Liver Disease Research Center, Iran University of Medical Sciences, Tehran, Iran; 4 Social Determinants of Health Research Center, Research Institute for Health Development, Kurdistan University of Medical Sciences, Sanandaj, Iran; 5 Department of Virology, Tehran University of Medical Sciences, Tehran, Iran; 6 Preventive Medicine and Public Health Research Center, Iran University of Medical Sciences, Tehran, Iran; 7 Cancer Theme SEC Faculty, Kingston University, Penrhyn Road, London, KT1 2EE UK; 8 Department of Social Medicine, Zanjan University of Medical Sciences, Zanjan, Iran; 9 Department of Epidemiology, School of Public Health, Iran University of Medical Sciences, Tehran, Iran; 10 School of Medicine, Shahid Beheshti University of Medical Sciences, Tehran, Iran

**Keywords:** Cancer, Genetics, Molecular biology

## Abstract

The present study was conducted to evaluate the prevalence of the signaling pathways mutation rate in the Gastrointestinal (GI) tract cancers in a systematic review and meta-analysis study. The study was performed based on the PRISMA criteria. Random models by confidence interval (CI: 95%) were used to calculate the pooled estimate of prevalence via Metaprop command. The pooled prevalence indices of signal transduction pathway mutations in gastric cancer, liver cancer, colorectal cancer, and pancreatic cancer were 5% (95% CI: 3–8%), 12% (95% CI: 8–18%), 17% (95% CI: 14–20%), and 20% (95% CI: 5–41%), respectively. Also, the mutation rates for Wnt pathway and MAPK pathway were calculated to be 23% (95% CI, 14–33%) and 20% (95% CI, 17–24%), respectively. Moreover, the most popular genes were APC (in Wnt pathway), KRAS (in MAPK pathway) and PIK3CA (in PI3K pathway) in the colorectal cancer, pancreatic cancer, and gastric cancer while they were beta-catenin and CTNNB1 in liver cancer. The most altered pathway was Wnt pathway followed by the MAPK pathway. In addition, pancreatic cancer was found to be higher under the pressure of mutation compared with others based on pooled prevalence analysis. Finally, APC mutations in colorectal cancer, KRAS in gastric cancer, and pancreatic cancer were mostly associated gene alterations.

## Introduction

Cell signaling is a communication process of cell activities mediated by downstream genes and proteins. Distraction of signaling process induce disturbance in cellular mechanisms and may cause diseases, such as cancer, autoimmunity, and diabetes. In the major category, the signaling pathways are divided into intracellular activating signaling pathways, such as Hippo signaling and Notch signaling pathways or the extracellular activating pathways, for instance, Mitogen-activated protein kinase (MAPK) signaling, Nuclear factor κB (NF-κB), Janus kinase^1^/signal transducer and activator of transcription (STAT) signaling pathway, Wnt signaling pathways, Hedgehog, Smad signaling pathway, and PtdIns 3-kinase (PI3) signaling pathways. The Smad signaling is critical in TGF-β signaling, which controls the transcription. MAPK signaling pathway makes use of three different downstream effectors, including Extracellular-signal-regulated kinase pathway, c-Jun N-terminal kinase (JNK) pathway, and p38 pathway. Also, the Wnt signaling pathway is important in cell differentiation and proliferation. In Wnt signaling, the Wnt/β-catenin signaling pathway is the only canonical pathway^2^. The p53 signaling is not a canonical signaling pathway but due to the p53 non-transcriptional functions, the role of this pathway in generating cancer and its interaction with other signaling pathways, p53 can be considered as an individual pathway^3^.

Gastrointestinal (GI) cancers are a group of cancers that affect the digestive system and its accessory organs. The most prevalent cancers related to GI tract are colorectal, gastric, and esophageal cancers, respectively^4^. Mutations in signaling pathways, such as signal transduction systems, are the basic triggering mechanisms in different types of cancers^5^. The role of MAPK signaling pathway, Wnt, TGF beta, and JAK-STAT signaling pathways are more common in cancer induction. The Wnt signaling pathway, which include genes like PTEN (phosphatase and tensin homolog deleted on chromosome 10), WISP3 (Wnt1-inducible signaling protein 3), APC (Adenomatous polyposis coli), β-catenin, AXIN, and TCF4 (T-cell factor 4), has significant role in carcinogenesis. Thus, its microsatellite instability (MSI), among other carcinogenesis processes, has been a hot topic, especially in the studies of colorectal cancer^6–8^. APC mutation and promoter hypermethylation are two important mechanisms in carcinogenesis and colorectal cancer (CRC) progression^9–11^. Two AXIN genes, AXIN1 and AXIN2, could be prone to mutation in some CRC cases^12,13^. PIK3CA (phosphatidylinositol-4, 5-bisphosphate 3-kinase catalytic subunit alpha) and PTEN are two important genes in the PI3K/AKT signal pathway and previous studies have put emphasis on them as important genes in the CRC development by altering the proliferation and cell death patterns^14,15^. Moreover, CTNNB1 (catenin beta 1) transformation via β-catenin alteration as another mediators of the Wnt/β-catenin pathway have been found in some of the liver tumors^16^. Liver carcinogenesis process is related to the interactions of three major pathways: the p53/p21, the p16/cyclin D1/pRB, and the Wnt/wingless^17,18^. Also, numerous factors such as TNFα (tumour necrosis factor alpha), TGFβ (transforming growth factor beta), c-myc, IGF2R (insulin like growth factor 2 receptor), SMAD2, SMAD4, DLC-1, and HIC1 (HIC ZBTB transcriptional repressor 1) could initiate liver tumorogenesis^17,18^.

Mutation analysis of signaling pathway mediators could have prognostic impact on tumor development. Transformation of the epidermal growth factor receptor (EGFR) and its downstream pathway mediators could lead to development of human tumors^19^. Two vital intracellular pathways affected by EGFR are the RAS/RAF/MAPK and the PIK3CA/PTEN/AKT signaling pathways. These pathways mediate activation of transcription factors like ERK (extracellular regulated MAP kinase) and p38 and lead to cell transformation reactions like the up-regulation of proliferation, relocation, mesenchymal separation induction, and apoptosis reduction. As EGFR has been a target for anti-tumor drugs, its mutations and related downstream signaling pathway mutations have become important^20^.

Indeed, interaction of various signaling pathway mediator mutations and their behavior in cancer development has been a hot topic. These alterations could include susceptibility, resistant or non-sense for treatment management or tumorogenesis in different individuals geographically. By considering the PRISMA (Preferred Reporting Items for Systematic Reviews and Meta-Analyses) criteria^21^, we made an attempt to evaluate the prevalence of the signaling pathways mutation rate in the GI tract cancers in a systematic review and meta-analysis setting.

## Results

### Search results

**Figure 1:**
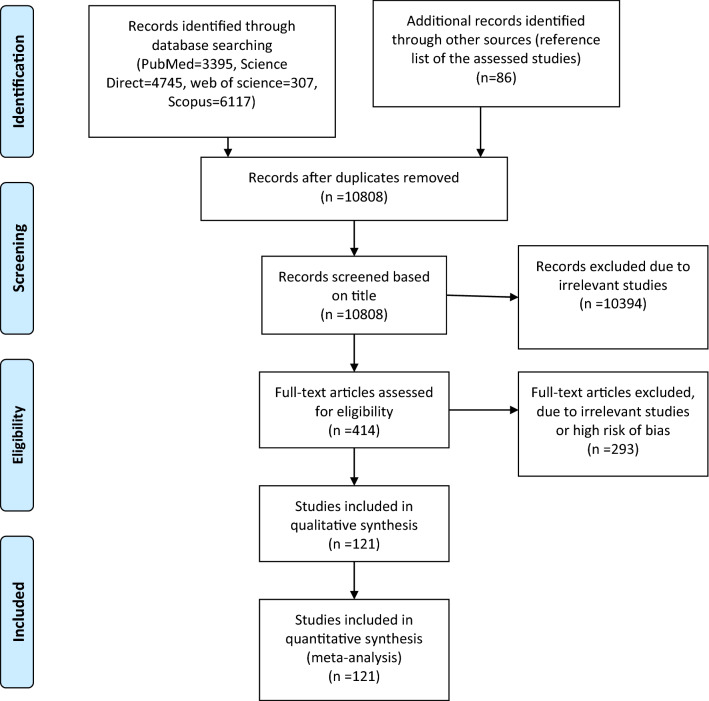
PRISMA Flow Diagram of our study population, the diagram indicates the primary search item frequencies, duplicates, Studies included in qualitative synthesis and Studies included in quantitative synthesis.

A total of 10,808 records were detected using the search strategy keywords. After screening by the title and abstracts, 414 articles were included for further analysis. Next, the full-text assessment resulted in selecting 121 eligible records including 65 studies on colorectal cancer (CRC), 21 on liver cancer (LC), 16 on Gastric Cancer (GC), 9 on pancreatic cancer^1^, and 15 on other gastrointestinal cancers, namely esophagus, bile duct, rectal cancer, gall bladder, and ampullary adenocarcinomas. The details of screened data based on PRISMA guideline are provided in Fig. 1. The numbers of participants for the assessment of the GI cancer mutations induced 17,269, 1056, 2500, 378, 1080 individuals for CRC, LC, GC, PC, and other GI cancers, respectively.

### Bias assessment

The risk of bias assessment is given in Table 1. Also, the RTI tool for the risk of bias determined one study with high risk of Selection Bias. Also, the Selection Bias, Performance Bias, Detection Bias, and Selective Outcome bias indicated 25, 3, 4, and 33 studies with unclear risk of bias, respectively. Furthermore, high risks of Selection Bias and Selective Outcome Bias were evaluated in 3 and 2 references, respectively.

**Table 1:** Key: + : Low risk of bias, − High risk of bias ?, Unclear risk of bias, *: Non-applicable in non RCT by RTI.

	Author	Year	Country	Selection bias	Performance bias	Detection bias	Attrition bias	Selective outcome	Confounding	Ref
1	Müller	1998	Germany	?	?	+	*	+	+	^22^
2	Sparks	1998	USA	−	?	+	*	+	+	^23^
3	Kondo	1999	Japan	−	+	+	*	+	+	^16^
4	Koyama	1999	Japan	?	+	+	*	?	+	^24^
5	Shitara	1999	Japan	+	+	+	*	?	+	^25^
6	Mirabelli	1999	Canada	+	+	+	*	+	+	^26^
7	Huang	1999	France	+	+	+	*	+	+	^27^
8	Wong	2001	China	+	+	+	*	+	+	^28^
9	Fujimori	2001	Japan	+	+	+	*	+	+	^29^
10	Kawate	2001	Japan	?	+	+	*	?	+	^30^
11	Rashid	2001	China	+	+	+	*	+	+	^31^
12	Shitoh	2001	Japan	+	+	+	*	+	+	^32^
13	Chen	2002	Taiwan	?	+	+	*	?	+	^33^
14	Taniguchi	2002	United States	+	+	+	*	+	+	^34^
15	Clements	2002	USA	+	+	+	*	?	+	^35^
16	Engeland	2002	Netherlands	+	+	+	*	+	+	^36^
17	Yuen	2002	UK	?	+	+	*	+	+	^37^
18	Abraham	2002	United States	?	+	+	*	+	+	^38^
19	Yoo	2002	South Korea	+	+	+	*	+	+	^39^
20	Tannapfel	2003	Germany	?	+	+	*	+	+	^40^
21	Jass	2003	Australia	+	+	+	*	+	+	^41^
22	Zhang	2003	Japan	+	+	+	*	+	+	^42^
23	Sakamoto	2004	Japan	+	+	+	*	?	+	^43^
24	Bläker	2004	Germany	?	+	+	*	?	+	^44^
25	Fransén	2004	Sweden	+	+	+	*	+	+	^45^
26	Li	2005	China	+	+	+	*	+	+	^46^
27	Immervoll	2005	Norway	+	+	+	*	−	+	^47^
28	Pasche,	2005	USA	+	+	+	*	+	+	^48^
29	Thorstensen	2005	Norway	+	+	+	*	+	+	^49^
30	Noda	2006	Japan	+	+	?	*	?	+	^50^
31	Mikami	2006	Japan	+	+	+	*	+	+	^51^
32	Schönleben	2008	USA	+	+	+	*	?	+	^52^
33	Ching-Shian Leong,	2008	Malaysia	+	?	?	*	?	+	^53^
34	Nomoto	2008	Japan	?	+	+	*	+	+	^54^
35	Schonleben	2008	Germany	?	+	+	*	+	+	^55^
36	Pan	2008	China	+	+	+	*	+	+	^56^
37	Kim	2008	Korea	+	+	+	*	+	+	^57^
38	Xie	2009	Korea	+	+	+	*	+	+	^58^
39	Seth	2009	UK	−	+	+	*	+	+	^59^
40	Cieply	2009	USA	+	+	+	*	+	+	^60^
41	Dahse	2009	Germany	+	+	+	*	+	+	^61^
42	Kim	2009	South Korea	+	+	+	*	+	+	^62^
43	Packham	2009	Australia	+	+	+	*	?	+	^63^
44	Baldus	2010	Germany	+	+	+	*	+	+	^64^
45	Irahara	2010	USA	+	+	+	*	+	+	^65^
46	Smith	2010	UK	+	+	+	*	?	+	^66^
47	Liao	2010	China	?	+	+	*	?	+	^67^
48	Catenacci	2011	USA	+	+	+	*	+	+	^68^
49	Watanabe	2011	Japan	+	+	+	*	+	+	^69^
50	Metzger	2011	Luxembourg	+	+	+	*	?	+	^70^
51	Naghibalhossaini	2011	Iran	+	+	+	*	−	+	^71^
52	Sameer	2011	India	+	+	+	*	+	+	^72^
53	Purcell	2011	New Zealand	+	+	+	?	+	+	^73^
54	Ueda	2011	Japan	+	+	+	*	+	+	^74^
55	Mohri	2012	Japan	?	+	+	*	+	+	^75^
56	Sukawa	2012	Japan	+	+	+	*	+	+	^76^
57	Bond	2012	Australia	+	+	+	?	+	+	^77^
58	Laghi	2012	Italy	+	+	+	*	+	+	^78^
59	Levidou	2012	Greece	+	+	+	*	+	+	^79^
60	Lee	2012	Korea	+	+	+	*	+	+	^80^
61	Li	2012	China	+	+	+	*	?	+	^81^
62	Paliga	2012	Canada	+	+	+	*	?	+	^82^
63	Voorham	2012	Netherlands	+	+	+	*	+	+	^83^
64	Whitehall	2012	Australia	+	+	+	*	+	+	^84^
65	Khiari	2012	Tunisia	+	+	+	*	?	+	^85^
66	Tai	2012	Taiwan	+	+	+	*	+	+	^86^
67	Ree	2012	Norway	+	+	+	*	+	+	^87^
68	Chen	2013	Taiwan	+	+	+	*	?	+	^88^
69	Garcia-Carracedo	2013	USA	?	+	+	*	+	+	^89^
70	Hidaka	2013	Japan	+	+	+	*	?	+	^90^
71	Kan	2013	USA	+	+	+	*	+	+	^91^
72	Saigusa	2013	Japan	+	+	+	*	+	+	^92^
73	Shi	2013	China	?	+	+	*	?	+	^93^
74	Aissi	2013	Tunisia	+	+	+	*	?	+	^94^
75	Fleming	2013	USA	+	+	+	*	+	+	^95^
76	Long	2013	China	+	+	+	*	+	+	^96^
77	Van Grieken	2013	UK, Japan, Singapore	+	+	+	*	?	+	^97^
78	Gurzu	2013	Romania	+	+	+	*	+	+	^98^
79	Wang	2013	USA	+	+	+	*	+	+	^99^
80	Han	2013	Korea	+	+	+	*	?	+	^100^
81	Neumann	2013	Germany	+	+	+	*	+	+	^101^
82	Shen	2013	China	+	+	+	*	+	+	^102^
83	Yip	2013	Malaysia	?	+	+	*	+	+	^103^
84	Zhang	2014	China	+	+	+	*	+	+	^104^
85	Mohammadi asl	2014	Iran	+	+	+	*	?	+	^105^
86	Chen	2014	China	+	+	+	*	+	+	^106^
87	Lee	2014	Korea	+	+	+	*	?	+	^107^
88	Ahn	2014	Korea	+	+	+	*	?	+	^108^
89	Chang	2014	Taiwan	?	+	+	*	+	+	^109^
90	Jia	2014	China	?	+	?	*	?	+	^110^
91	Wang	2014	USA, China	+	+	+	*	+	+	^111^
92	Zhu	2014	China	+	+	+	*	+	+	^112^
93	Tong	2014	PR China	+	+	+	*	+	+	^113^
94	Gao	2014	China	+	+	+	*	?	+	^114^
95	Li	2014	China	?	+	+	*	+	+	^115^
96	Saito	2014	Japan	?	+	+	*	+	+	^116^
97	Schlitter	2014	Germany	?	+	+	?	+	+	^117^
98	Marchio	2014	Peru	+	+	+	*	+	+	^118^
99	Mikhitarian	2014	USA	?	+	+	*	+	+	^119^
100	Yoda	2015	Japan	?	+	+	*	+	+	^120^
101	Zaitsu	2015	Japan	+	+	+	*	+	+	^121^
102	Lu	2015	China	?	+	+	*	?	+	^122^
103	Kawamata	2015	Japan	+	+	+	*	?	+	^123^
104	Lan	2015	Taiwan	+	+	+	*	+	+	^124^
105	Samara	2015	Greek	+	+	+	*	+	+	^125^
106	Abdelmaksoud Damak	2015	Tunisia	+	+	+	*	?	+	^126^
107	Kawazoe	2015	Japan	+	+	+	*	+	+	^127^
108	Lin	2015	USA	+	+	+	*	+	+	^128^
109	Suarez	2015	France	+	+	+	*	?	+	^129^
110	Witkiewicz	2015	USA	+	+	+	*	+	+	^130^
111	Okabe	2016	USA	+	+	+	*	+	+	^131^
112	Grellety	2016	France	+	+	+	*	?	+	^132^
113	Jauhri	2016	India	+	+	+	*	?	+	^133^
114	Nam	2016	Republic of Korea	+	+	+	*	+	+	^134^
115	Dallol	2016	Saudi Arabia	+	+	+	*	+	+	^135^
116	Yuan	2016	China	?	+	+	*	+	+	^136^
117	Ziv	2017	New York	?	+	?	*	+	+	^137^
118	Ho	2017	Hong Kong	+	+	+	*	+	+	^138^
119	Hänninen	2018	Finland	+	+	+	*	+	+	^139^
120	Mizuno	2018	USA	+	+	+	*	+	+	^140^
121	Yang	2018	China	+	+	+	*	+	+	^141^

### Signaling pathways mutations in gastric cancer

From among 16 studies on GC, mostly the MAPK and PI3 pathways were analyzed in 2489 participants. The most evaluated gene in MAPK was KRAS and mutations ranged from 0 to 20%. Also, the PI3K mutations in the PI3 pathway were 3 to 8.7% and CTNNB1 mutations ranged from 1.7% to 7%. The detailed data are listed in Table 2 and supplementary Table 2.

**Table 2:** GI tract cancer signaling pathway mutations based on genes and exon (n = 121).

Cancer type (number of studies)	Pathway (number of studies)	Gene (number of studies)	Exon	Mutant%	Sample No	Reference(s)
CRC (n = 65)	MAPK (n = 43)	KRAS (n = 46)	1	24	86	^142^
			1, 2	14.6	48	^43^
			2	34–44.9	1167	^64,101,106,125,127,141^
			2, 3, 4	49	37	^59^
			3, 4	3.8	264	^127^
			NR	2.5–75	11,561	^36,42,45,50,51,63,65–67,69,71,77,79,83,84,86,92,94,98,99,102,103,107–109,112,113,123,124,128,132,134,135^
		BRAF (n = 33)	NR	0–78	8146	^37,45,50,51,63,65,67,71,77–79,83,84,93,98,108,109,112,127,128,132,134^
			11, 13–15	10	37	^59^
			11, 15	6.9	676	^102^
			15	2.3–46.2	982	^64,79,101,103,105,106,125,141^
	Wnt (n = 18)	beta-catenin (n = 6)	3	3–37.5	491	^26,29,32,51^
			NR	4–27	97	^22,42^
		APC (n = 10)	NR	28–73	750	^41,83,88,99,107,128,135^
			15	50–52	180	^32,126^
		AXIN2 (n = 2)	7, 8	1.4–20	381	^49,62^
		CTNNB1 (n = 7)	3	1.3–16	274	^85,126^
			NR	1–48	387	^23,83,128,133^
	PI3 (n = 15)	PIK3CA (n = 17)	9, 22	0–21	1556	^51,53,64,67,101,102,106^
			NR	0–34	3634	^65,83,84,107,109,112,124,127,128,134,135^
		PTEN (n = 7)	1–9	0	49	^103^
			8	17	310	^49^
			NR	0–28	459	^83,128,133,135^
	P53 (n = 5)	P53 (n = 5)	NR	18–63	1589	^49,77,99,128,135^
LC (n = 21)	MAPK (n = 3)	KRAS (n = 3)	2–18	0	25	^40^
			NR	4–16	92	^118,122^
		BRAF (n = 2)	NR	0	105	^40,118^
	Wnt (n = 15)	beta-catenin (n = 8)	NR	15–70	225	^33,34,91,129^
			3	2.8–41	156	^16,27,28,57^
		AXIN (n = 3)	3–5	25	36	^57^
			NR	2–12.5	153	^34,118^
		CTNNB1 (n = 7)	3	12–75	370	^34,60,73,74,131^
			NR	15–31	86	^110,118^
	P53 (n = 4)	TP 53 (n = 4)	NR	1.2–61	296	^91,96,118,122^
PC (n = 9)	MAPK (n = 5)	KRAS (n = 6)	1	47–67	79	^47,55^
			2	27	11	^75^
			NR	42–92	199	^52,119,130^
		BRAF (n = 4)	5, 11, 15	0–2.7	79	^47,55^
			NR	0–2.7	90	^52,119^
	Wnt (n = 2)	beta-catenin (n = 1)	3	23	21	^38^
		AXIN (n = 1)	NR	5	109	^130^
	PI3 (n = 4)	PIK3CA (n = 5)	All	11	36	^55^
			NR	4–11	147	^52,130^
			9	12	52	^119^
			9, 20	2.7	36	^89^
GC (n = 16)	MAPK (n = 5)	KRAS (n = 4)	1	14	104	^39^
			2	0	34	^141^
			NR	4.2–20	767	^97,120^
	Wnt (n = 6)	AXIN1 (n = 2)	NR	3.8–7.1	200	^56,90^
		AXIN2 (n = 3)	NR	4.6–9.8	292	^56,62,90^
		APC (n = 1)	NR	2.5	237	^80^
		CTNNB1 (n = 4)	NR	1.7–3.6	322	^80,90,120^
			3	7.1	70	^56^
	PI3 (n = 5)	PIK3CA (n = 5)	NR	5.1–7.2	292	^80,120^
			1, 9, 20	4.3–8.7	325	^46,76^
			18	3	100	^104^
		PTEN (n = 1)	NR	20	221	^121^
		AKT (n = 1)	6	2	100	^104^

*NR* not reported.

**Figure 2:**
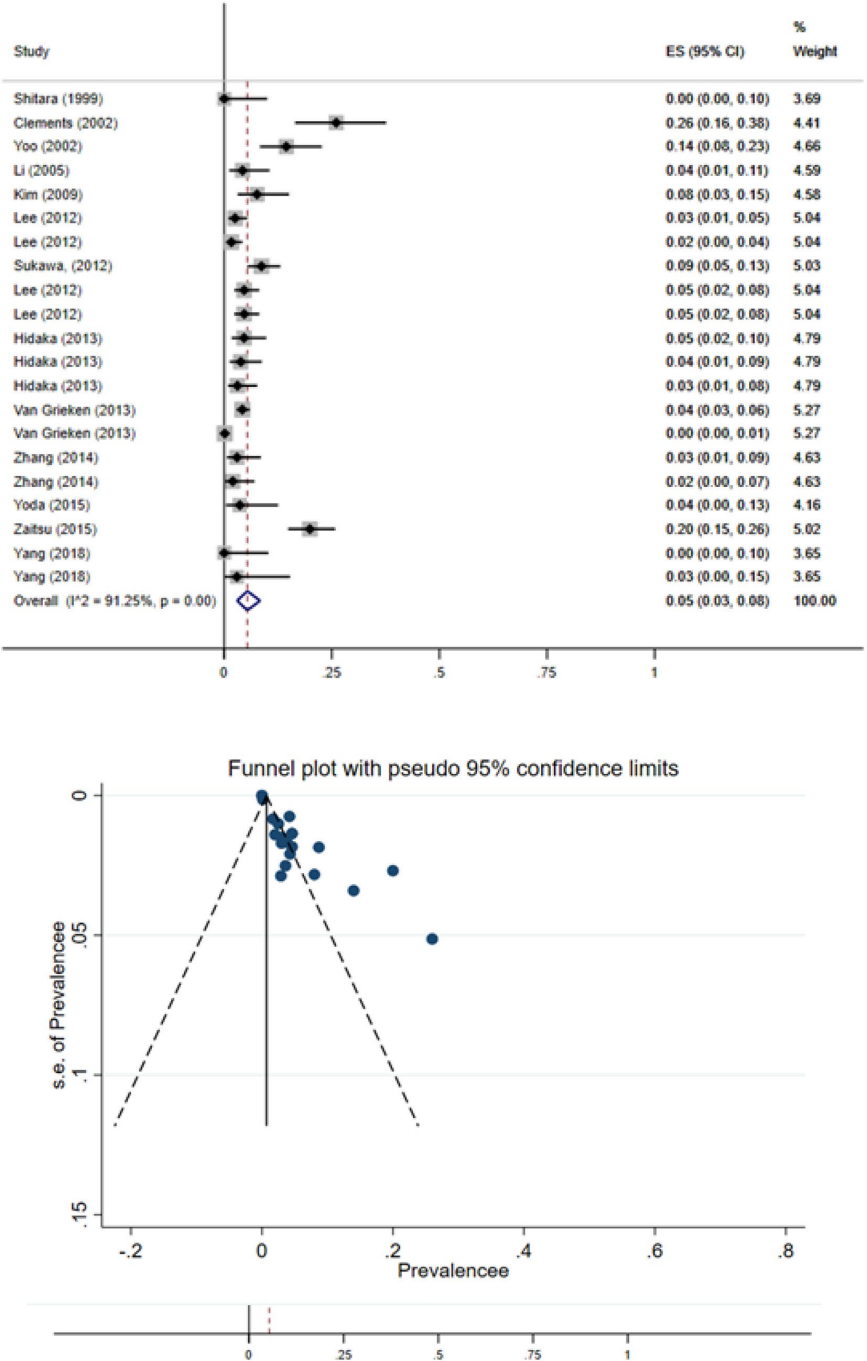
Heterogeneity and pooled prevalence funnel plot of the included studies for GC signal transduction pathway mutations.

The results of meta-analysis revealed that pooled prevalence index of signal transduction pathway mutations in GC was 5% (95% CI: 3–8%) and there was high heterogeneity between these studies in estimating the prevalence (I-squared = 91.25%, *P* = 0.001) (Fig. 2). Also, since the CI of the test (Egger’s test) does not include zero, there is no bias in our results (Egger's test = 3.51, *P* = 0.0001, 95% CI: 2.49 to 4.53). The pooled prevalence funnel plot in GC signal transduction pathway mutations is illustrated in Fig. 2. Furthermore, the Subgroup analyses of pooled prevalence Signal Transduction Pathway Mutations in GC are summarized in Table 3.

**Table 3:** Subgroup analysis of pooled prevalence of Signal Transduction Pathway Mutations in GC, CRC, HCC, and PC based on gene, pathway, and method of diagnosis.

Outcome	Subgroup	No. of studies	Summery Odds Ratio (95% CI)	Between studies		
				I^2^	P _heterogeneity_	Q
GC	**Gene**					
	AXIN2 CTNNB1 KRAS BRAF PIK3C	2 3 4 2 4	6% (3– 9%) 2% (1–4%) 14% (2–34%) 0% (0–0%) 5% (3–8%)	7.7% 0.0% 96.3% 39.2% 41.43%	0.298 0.592 0.001 0.200 0.160	3.78 3.19 8.15 1.42 6.38
	**Pathway**					
	Wnt MAPK PI3	8 5 6	5% (2–9%) 7% (1–17%) 6% (2–12%)	83.4% 95.3% 88.7%	0.0001 0.0001 0.0001	5.03 2.84 4.50
	**Method for detection**					
	PCR, SS Array ARMS-PCR PCR-SSCP	13 4 2 2	8% (4–14%) 3% (2–5%) 1% (0–6%) 4% (1–9%)	94.7% 40.0% 29.0% 40.43%	0.0001 0.170 0.130 0.345	5.33 7.37 1.00 3.65
CRC	**Gene**					
	Beta-Catenin CTNNB1 APC KRAS BRAF NRAS SMAD4 PTEN PIK3C	4 5 7 41 27 6 6 5 17	17% (4–36%) 9% (1–22%) 44% (33–55%) 32% (29–36%) 9% (6–12%) 7% (0–23%) 7% (3–12%) 5% (0–14%) 9% (6–12%)	92.97% 93.35% 89.18% 94.24% 95.83% 99.17% 90.65% 90.97% 92.65%	0.001 0.001 0.001 0.001 0.001 0.001 0.001 0.001 0.001	3.30 2.94 11.68 29.60 9.22 5.24 5.03 10.48 14.07
	**Pathway**					
	Wnt MAPK/ERK Smad (TGF-β) PI3	18 73 9 21	23% (14–33%) 20% (17–24%) 7% (4–10%) 9% (6–12%)	96.25% 97.74% 86.69% 91.29%	0.001 0.001 0.001 0.001	7.69 19.68 7.51 10.58
	**Method for detection**					
	PCR, SS High-throughput Genotyping NGS PCR, Pyrosequencing	67 9 18 12	17% (14–21%) 4% (0–12%) 28% (22–35%) 17% (11–25%)	97.21% 95.90% 94.90% 96.95%	0.001 0.001 0.001 0.001	16.90 2.44 1.96 13.69
LC (HCC)	**Gene**					
	Beta-Catenin	7	20% (10–31%)	77.20%	0.001	6.06
	**Pathway**					
	Wnt	13	17% (11–23%)	72.34%	0.001	9.11
	**Method for detection**					2.56
	SSCP, SS PCR, SS	5 16	14% (1–34%) 11% (6–17%)	92.16% 79.51%	0.001 0.001	6.04 4.22
PC	**Gene**					
	KRAS	5	58% (31–83%)	93.64%	0.001	5.60
	PIK3C	4	6% (3–10%)	14.84%	0.320	5.13
	**Pathway**					
	MAPK	8	31% (5–66%)	97.66%	0.001	4.75
	PI3	4	6% (3–10%)	14.84%	0.320	5.13
	**Method for detection**					
	PCR, SS	11	31% (5–66%)	92.05%	0.001	3.84

GC: gastric cancer; CRC: colorectal cancer; HCC: hepatocellular carcinoma; PC: pancreatic cancer. SS: Sanger Sequencing, SSCP: Single-stranded conformation polymorphism; HPLC: High-performance liquid chromatography, NGS: next-generation sequencer, ARMS-PCR: amplification refractory mutation system polymerase chain reaction.

### Signaling pathways mutations in CRC

CRC related signaling pathway mutation was found in 65 studies. A majority of study samples had the mean age > 60 years and male/female ratios of CRC incidence in most of the evaluated studies were reported more than 2:1. The most prevalent mutation analysis was taken from KRAS exon 2, BRAF exon 15, PIK3CA exon 9 and 20, and APC and beta-Catenin exon 3. Most of the studies were cross-sectional and total CRC patients included 17,269 cases. These studies reported different mutation rates based on the sample size, selected gene, and method of use. The results showed a wide range of mutation in different pathways and related genes as listed in supplementary Table 3. The KRAS mutations in the MAPK pathway were 2.5 to 75% and the BRAF (B-Raf proto-oncogene, serine/threonine kinase) mutations ranged from 0 to 78.6%. The Wnt signaling mediator mutations, such as beta-catenin, were reported from 3 to 37.5% and APC mutations ranged from 28.4 to 73%. The p53 was assessed in 5 studies and its mutation rate was reported 18–65% (Table 2).

**Figure 3:**
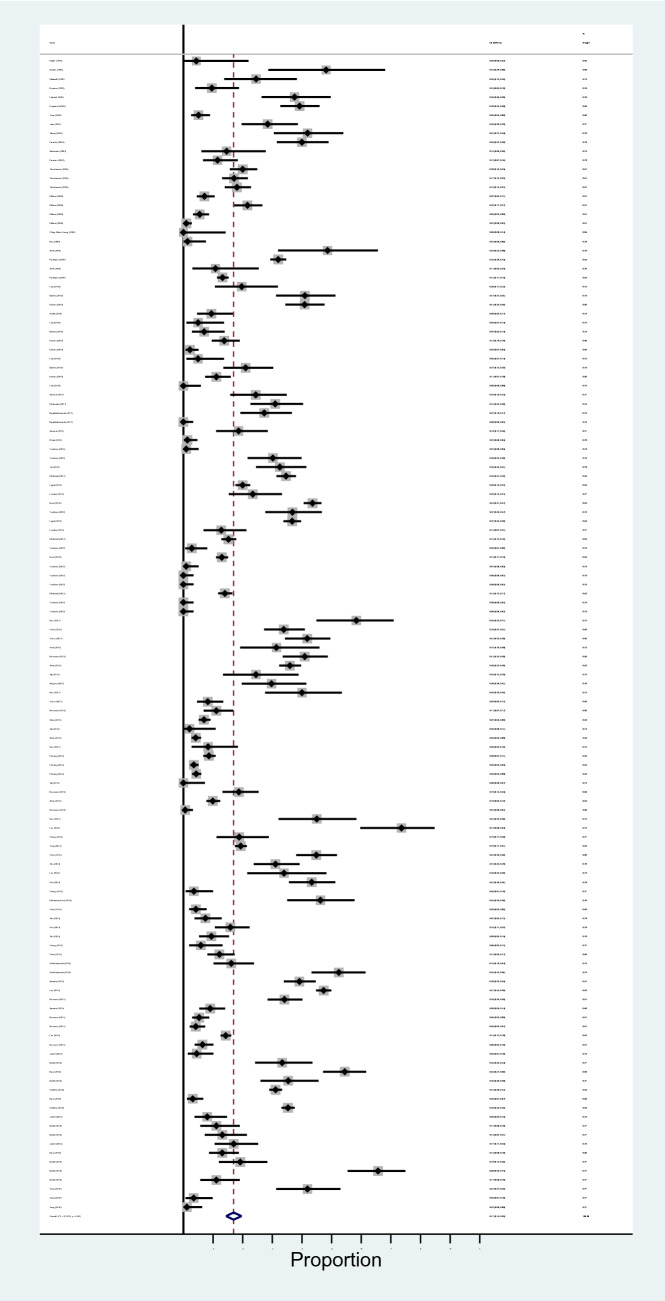
Heterogeneity plot of the included studies for CRC signal transduction pathway mutations.

**Figure 4:**
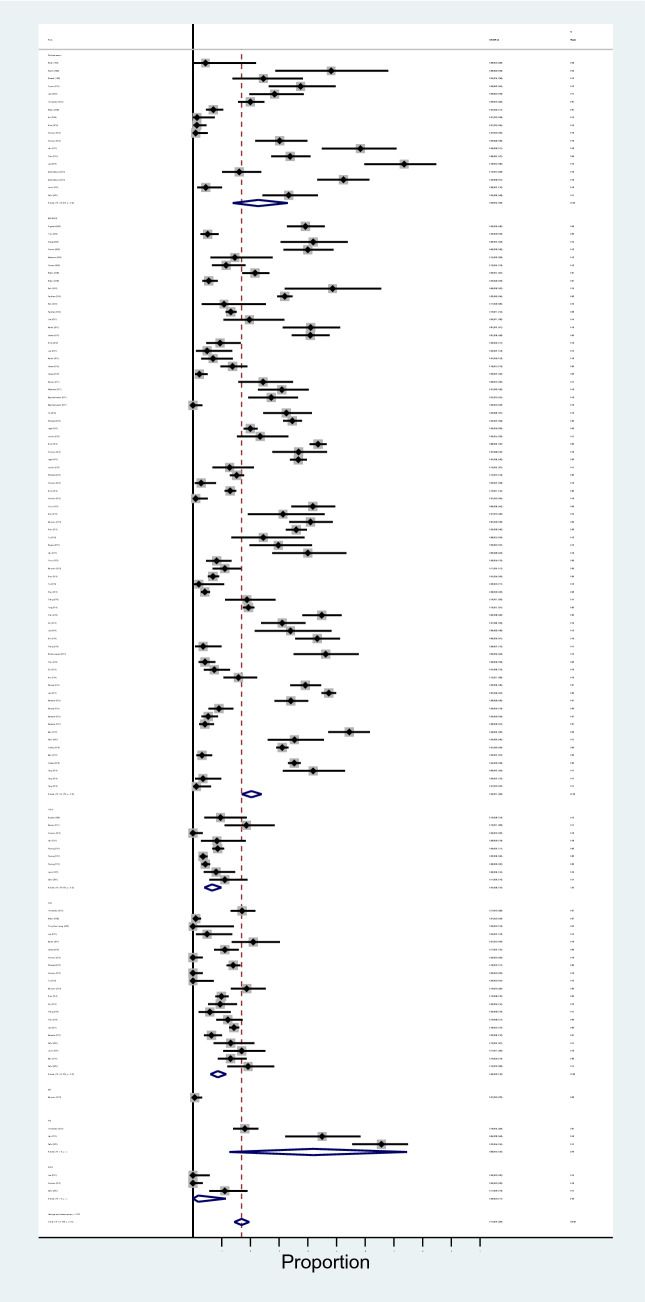
Subgroup analysis for heterogeneity based on the different pathways for CRC signal transduction pathway mutations.

**Figure 5:**
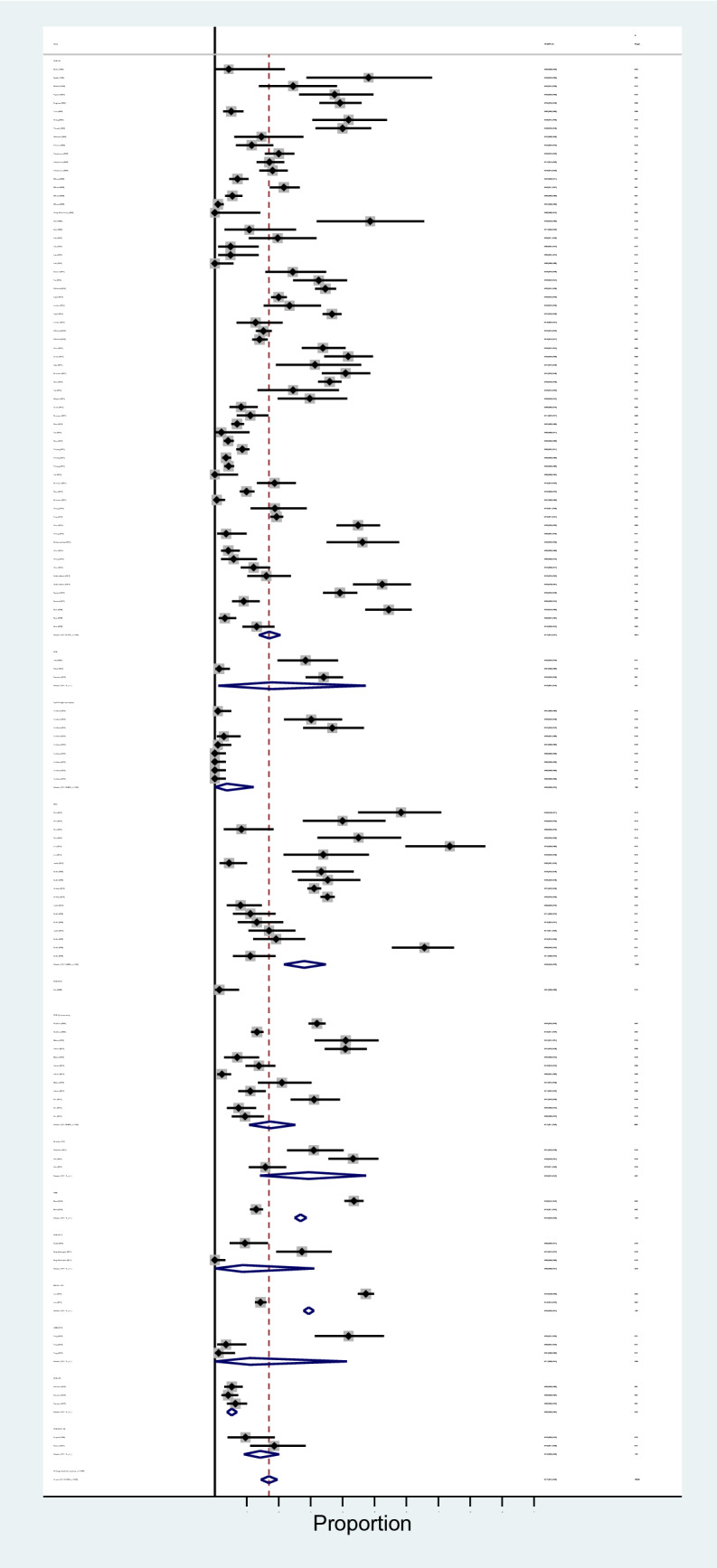
Subgroup analysis for heterogeneity based on the detection method for CRC signal transduction pathway mutations.

**Figure 6:**
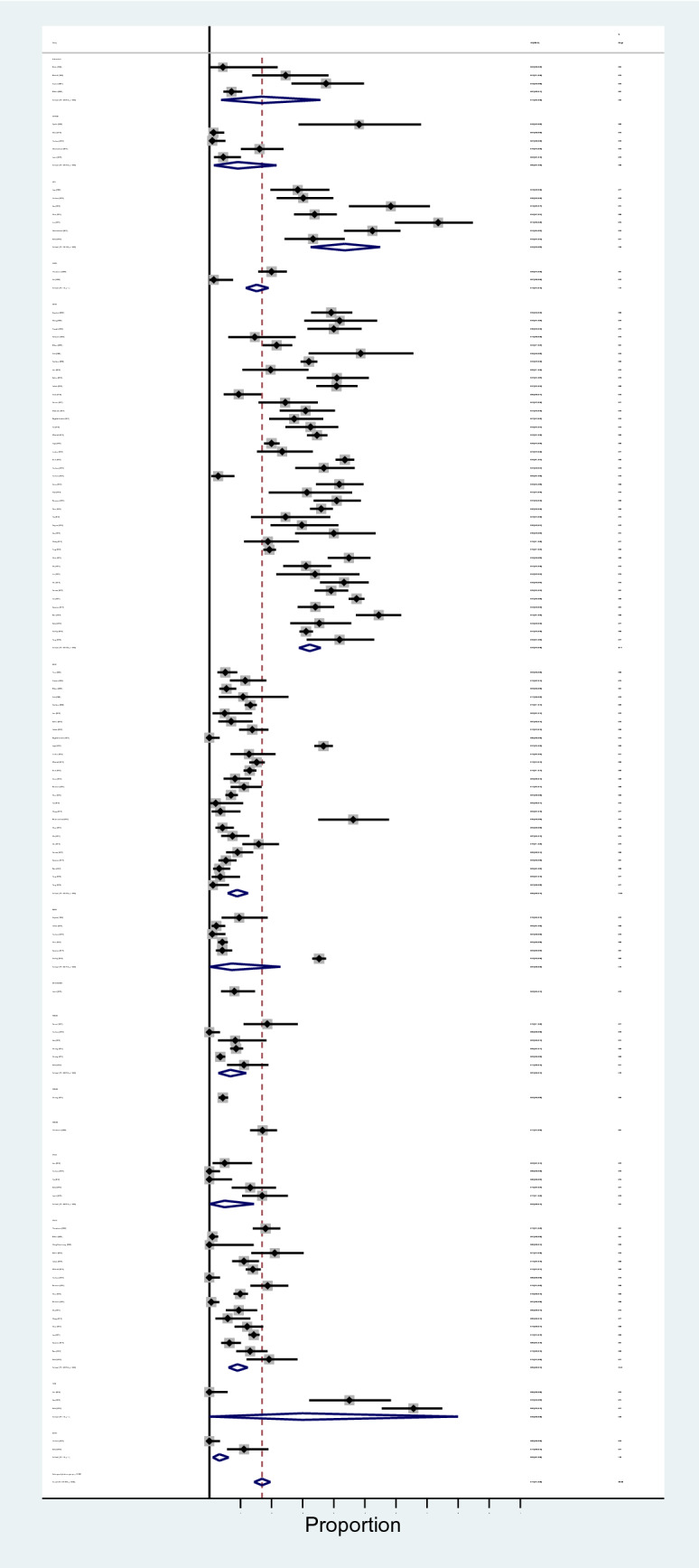
Subgroup analysis for heterogeneity based on involved genes for CRC signal transduction pathway mutations.

**Figure 7:**
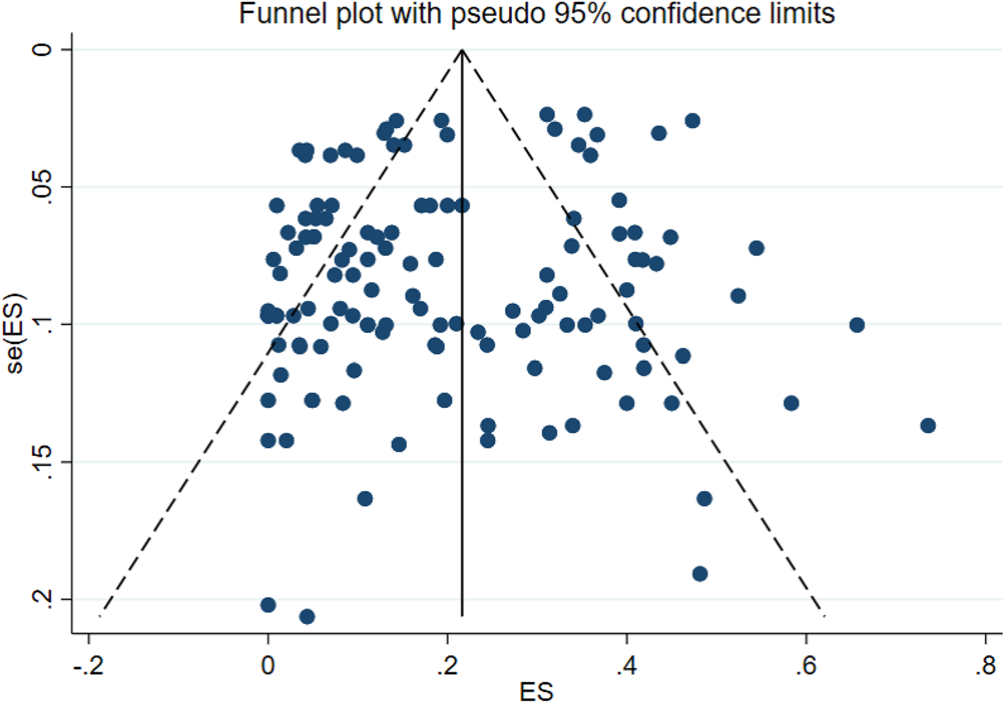
Pooled prevalence funnel plot in CRC signal transduction pathway mutations.

The results of meta-analysis revealed that pooled prevalence of signal transduction pathway mutations in CRC was 17% (95% CI: 14%, 20%) and there was a high heterogeneity between these studies in estimating the prevalence (I-squared = 97.63%, *P* = 0.0001) (Fig. 3). Also, the subgroup analysis for heterogeneity was performed in CRC included studies based on the different pathways (heterogeneity plot in Fig. 4), detection method (heterogeneity plot in Fig. 5), and involved genes (heterogeneity plot in Fig. 6). The CI of test (Egger’s test) included zero, thus there was no significant bias in the results (Egger's test = − 0.692, *P* = 0.109, 95% CI: − 1.54 to 0.156). The pooled prevalence funnel plot in CRC signal transduction pathway mutations is illustrated in Fig. 7 and the Subgroup analyses of pooled prevalence signal transduction pathway mutations in CRC are summarized in Table 3.

### Signaling pathway mutations in liver cancer (LC)

The search on liver cancer resulted in a total of 1056 hepatocellular carcinoma (HCC) and 174 hepatoblastoma participants in 21 studies. There were different ranges of mutations in these studies, which are listed in supplementary Table 4. The Wnt signaling was the most evaluated pathway in which the CTNNB1 gene mutation ranges were evaluated to be 12–75% and the beta-catenin genes had the mutation ranges of 2.8–41%. In addition, the mutation ranges in p53 were 1.2 to 61% and the JAKs in the JAK signaling pathway were observed to be 1.2 to 16%.

**Figure 8:**
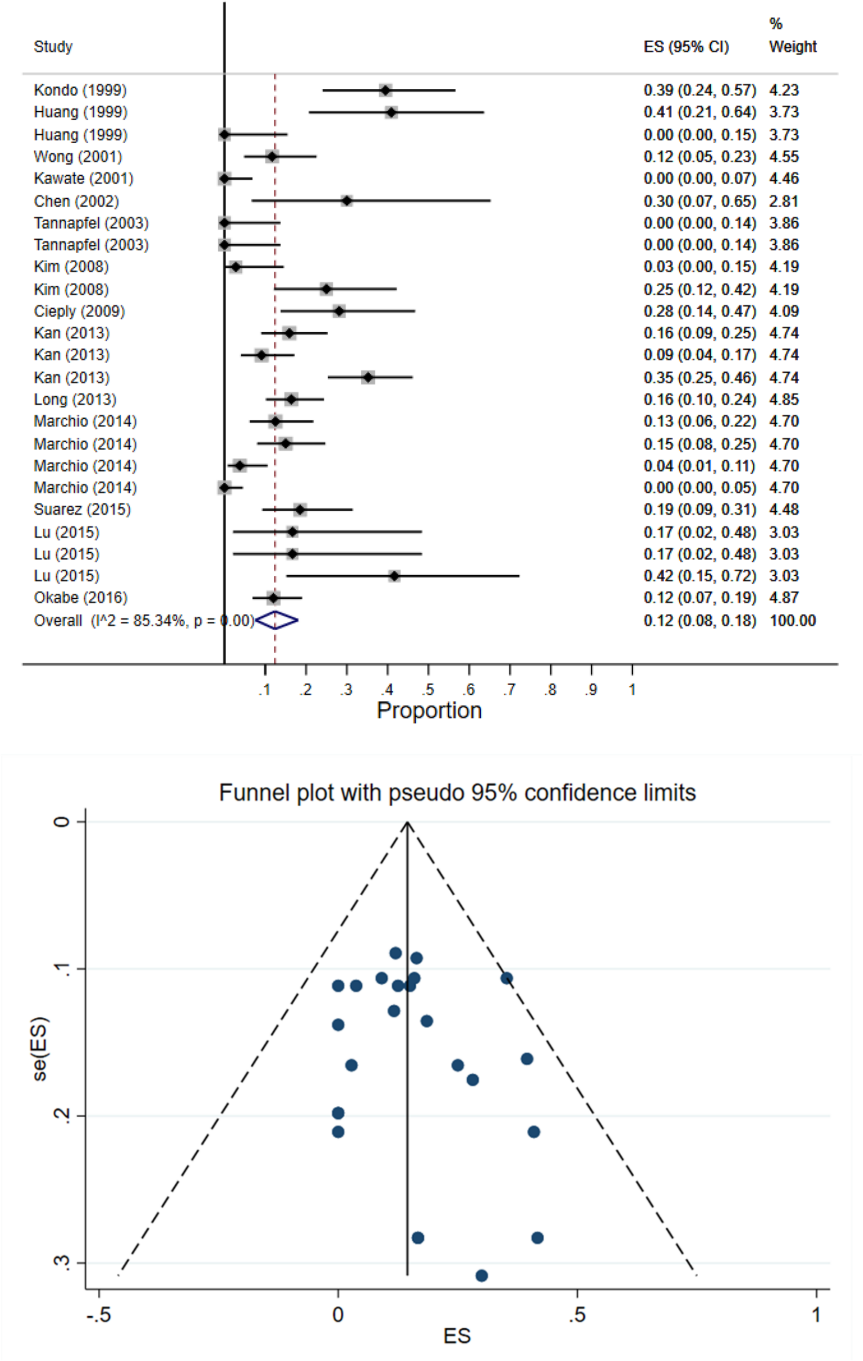
Heterogeneity and pooled prevalence funnel plot of the included studies for liver cancer signal transduction pathway mutations.

The results of meta-analysis showed that pooled prevalence of signal transduction pathway mutations in LC was 12% (95% CI: 8–18%) and there was a high heterogeneity between these studies in estimating the prevalence (I-squared = 85.34%, *P* = 0.0001) (Fig. 8). Also, since the CI of the test (Egger’s test) included zero, there was no significant bias in the results (Egger's test = − 0.442, *P* = 0.411, 95% CI: − 0.65 to 1.53). The pooled prevalence funnel plot in LC signal transduction pathway mutations is illustrated in Fig. 8. Furthermore, the Subgroup analyses of pooled prevalence signal transduction pathway mutations in LC are summarized in Table 3.

### Signaling pathways mutations in pancreatic cancer^1^

In a total of 9 studies, 392 PC patients were studied with the KRAS and PIK3CA mutations reported 42 to 92% and 2.7 to 12%, respectively. More data are shown in supplementary Table 5.

**Figure 9:**
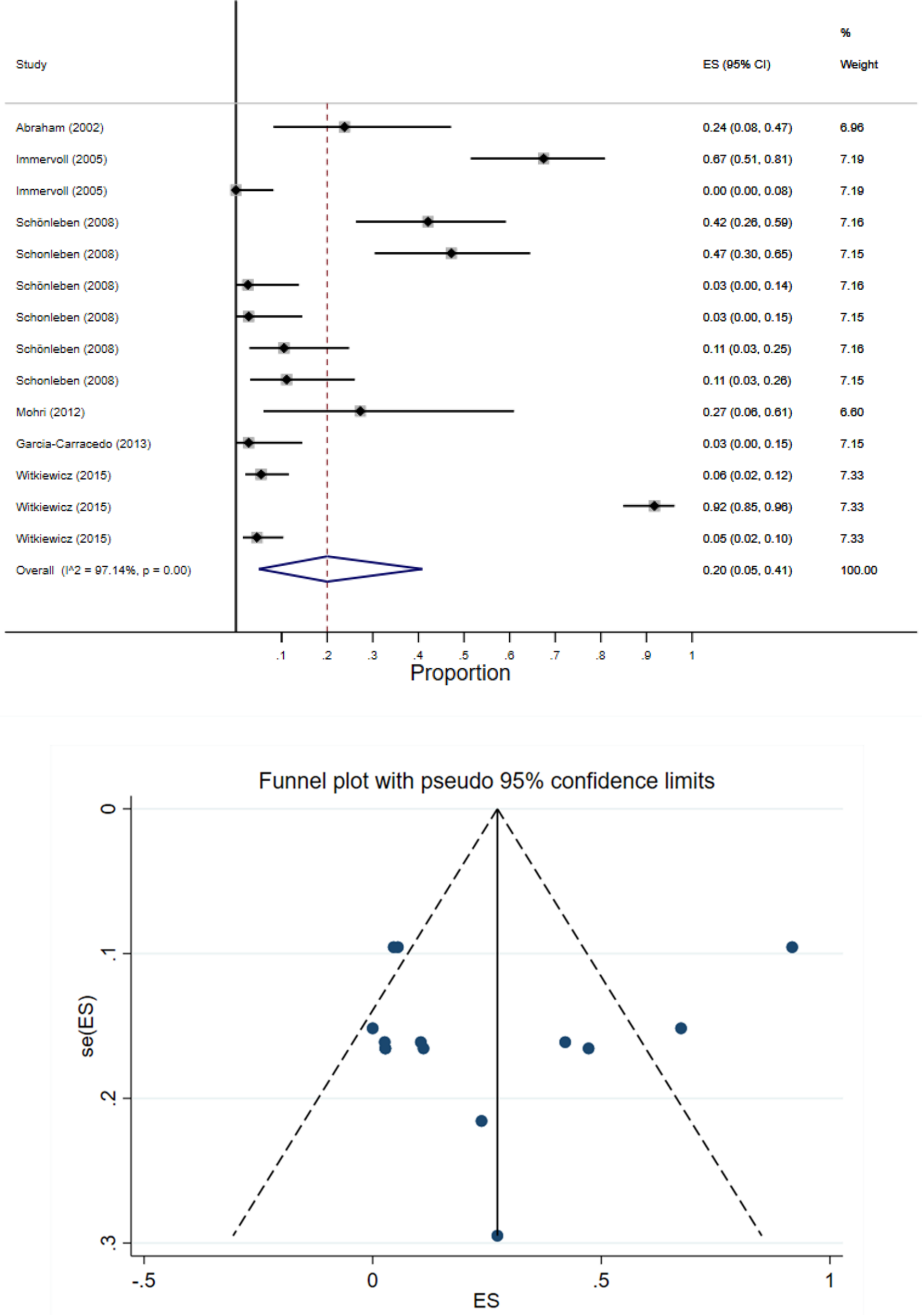
Heterogeneity and pooled prevalence funnel plot of the included studies for pancreatic cancer (PC) signal transduction pathway mutations.

The results of meta-analysis showed that pooled prevalence of signal transduction pathway mutations in pancreatic cancer was 20% (95% CI: 5–41%) and there was a high heterogeneity between these studies in estimating the prevalence (I-squared = 97.14%, *P* = 0.0001) (Fig. 9). Also, the CI of the test (Egger’s test) included zero, s no significant bias was present in the results (Egger's test = − 1.351, *P* = 0.568, 95% CI: − 6.37 to 3.66). The pooled prevalence funnel plot in PC signal transduction pathway mutations is illustrated in Fig. 9. Furthermore, the Subgroup analyses of pooled prevalence signal transduction pathway mutations in pancreatic cancer are summarized in Table 3.

### Signaling pathways mutations in other GI cancers

The other GI cancers included gastro-esophageal cancer, rectal cancer, esophageal squamous cell carcinoma, gallbladder carcinoma, and cholangiocarcinoma. Different signaling pathways in these GI cancers are listed in supplementary Table 6. Briefly, KRAS was the popular gene for mutation analysis ranging from 0% mutation in squamous cell anal carcinoma to 57% in small intestinal adenocarcinoma. BRAF was analyzed in 6 studies with its mutation reported to be 0–45%. Moreover, APC mutations were reported between 9.5 and 47% in different malignancies.

### Signaling pathway mutation association with clinic-pathological features and patients survival

The extracted data about clinic-pathological features and patients survival were listed in supplement Tables 2 to 6. As glimpse, the clinic-pathological features statistically significant in association with signaling pathway mutations that they were mentioned in 2 individual studies for gastric cancer and 30, 6, 1 and 2 individual studies for CRC, LC, PC and other GI cancers, respectively.

Survival rate assessment in association with signaling pathway mutations were listed in supplement Tables 2 to 6. The survival rate or prognostic feature in association with signaling pathway mutations were mentioned in 1, 6, 1, 1, 0 and 1 included studies for CRC, LC, PC and other GI cancers, respectively.

## Discussion

The aim of the current study was to evaluate the prevalence of the signaling pathway mutation rate in GI tract cancers in a systematic review and meta-analysis setting. It should note that, the signaling pathway mutations were comprehensively studied by The Cancer Genome Atlas (TCGA)^1^. Furthermore, the inclusion criteria for the current study were different with TCGA assessments. Also, this study could be a lead for further investigations in the field of the signaling pathway mutations prevalence and might be useful for further TCGA comprehensive updates. Appropriate keywords were used for search strategy in popular academic databases. Data were screened and eligibility of the studies was evaluated according to the inclusion criteria. PRISMA guideline was used as the study protocol. Through the search strategy, we found that GI malignancies included CRC, LC, PC, GC, esophageal cancer, rectal cancer, and bile duct neoplasm or cholangiocarcinoma. The results obtained in the current study showed that most alterations in CRC patients were in the KRAS gene in MAPK pathway within the range of 3.8 to 54.5%. These differences could be due to the study population or the methodology in different studies although the cancer stage and other risk factors could also play major roles. Furthermore, the pooled prevalence indices of signal transduction pathway mutations in GC, CRC, LC, and PC were 5% (95% CI: 3–8%), 17% (95% CI: 14–20%), 12% (95% CI: 8–18%), and 20% (95% CI: 5–41%), respectively. The higher rates in pooled prevalence could suggest more association between the signal transduction mutations and GI cancers incidence. The subgroup analysis for CRC shows that KRAS and APC are the most mutant genes with 32% (95% CI, 29–36%) and 44% (95% CI, 33–55%) mutation rates, respectively. Also, the most altered pathway was Wnt (23%) (95% CI, 14–33%), followed by MAPK (20%) (95% CI, 17–24%) pathway.

The CRC carcinogenesis is firstly initiated by the mild colon polyps and gradually progresses to the cancerous lesions. The adenocarcinoma is globally the most prevalent type of the CRC^143,144^. Recently, different studies have been reported focusing on the cost-effectiveness of the CRC screening programs indicating the importance of the CRC diagnosis^145,146^. Signaling pathways have crucial impacts on the development of different cancers^5^. Although the nucleotide alterations have critical impacts on cancer initiation, the environmental factors are predisposing elements in cancer induction and are affecting the signaling pathways mutations^147,148^. As an example, smoking affects CRC cancers generation and mortality^149–151^. In this regard, lung cancer investigations revealed that smoking could increase the EGFR and its downstream elements, such as KRAS and BRAF mutations^148^. Moreover, studies on CRC and smoking showed that TGFβ signaling pathway mutations have significant roles in carcinogenesis^147^. Inflammation is another key player in generation of cancer^152,153^. TLR2 alterations associated with inflammation could lead to the signaling pathways related ERK (extracellular-regulated kinase) and PI3K/AKT mutations. The importance of the inflammation in the CRC were illustrated by Liu and et al.^154^. These substitutions might be due to the microbiome disturbance, too^155^.

The MAPK/ERK signaling was analyzed in the study reported by Sameer et al.^156^ who found KRAS mutation to be 24% in 86 CRC patients. Meanwhile, Tong et al.^113^ reported the highest rate (75%) of the KRAS mutations in CRC patients in codon 12 in 1506 individuals. Tong’s study showed different mutation rates between the separate codons of the KRAS gene with the highest in codon 12 and the lowest (2.5%) in codon 61. Also, in the study conducted by Kawazoe et al.^127^ on 264 metastatic colorectal cancers (mCRC), the KRAS exon 2 mutation was calculated to be 34%, as the highest mutation rate. In this study, BRAF mutation rate was reported to be 5.4%. The highest prevalence for the BRAF mutation reported in other studies was 78%^88^. This huge difference in the BRAF mutation rate could be due to the differences in the sample size and the method used for analysis.

The Wnt/beta-catenin signaling and PI3K/AKT signal have been assessed in a variety of studies. The Wnt/beta-catenin was assessed in 18 different studies and the most evaluated genes were APC, beta-catenin, and CTNNB1. Fujimori et al.^26^ showed that 37.5% of the 73 CRC patients had mutations in the exon 3 of the beta-catenin gene. Also, Shitoh et al.^32^ reported the rate of 3% for beta-catenin mutation in exon 3, and 27% in the high-frequency microsatellite instability (MSI-H). Furthermore, the APC gene mutations were assessed in 10 different studies with the lowest reported to be 33% in the study by Chen^88^ study and the highest as 73% reported by Lee et al.^107^. The previous studies showed that the MSI could be associated with the in/del substitutions in genome hot spots which can initiate CRC tumorogenesis by increasing the mismatches indiscriminately^157–159^. Investigation on Wnt/beta-catenin signaling was firstly introduced by the association between APC gene and beta-catenin^160,161^. Other studies found the interactions of these genes with beta-catenin-Tcf (T-cell factor) complex suggesting the association of these genes with CRC omplication ^162^. The role of APC gene in causing cancer was initially introduced in the familial adenomatous polyposis (FAP)^163^. This gene facilitates beta-catenin distorting. APC gene mutations influence beta-catenin and AXIN protein binding sites^164,165^. Moreover, they could maximize the protein stability and life cycle^166^. Thus, the carcinogenesis process is accelerated by altered signal transduction and cell cycle^167^.

From among the studies which assessed the PI3 signal transduction pathway, the mutation of PIK3CA gene was reported in 20 studies ranging between 0 and 34%. Meanwhile, Thorstensen et al.^49^ found p53 gene mutation rate to be about 18% in CRC patients.

There are variable reports in the matter of clinic-pathological association with mutations in the current study. In the conducted study by Sameer et al.^156^ the clinic-pathological assessment indicated that, the SMAD4 mutations are more frequent in colon tumors and statistically associated with tumor grade and lymph nodes involvement. Tong and colleagues^113^ reports the KRAS mutations are in association with gender and tumor site. Also, Kawazoe et al.^127^ points out the BRAF mutations are associated with tumor location, site of metastasis and differentiation pattern. Meanwhile, Yang and colleagues^168^ reports the association of the KRAS mutations with tumor location, type of tumor, differentiation pattern and gender of the patients. Furthermore, there were limited data about the association of the mutations in signaling pathways with survival rate in patients. Some studies suggested BRAF mutations^169^ and SMAD4 mutations^140^ are association with poor prognosis and survival rate. Highly variable and limited data about clinic-pathological features, survival and prognosis in association with signaling pathway mutations were extracted. The clinic-pathological features and patients survival association with signaling pathway mutations is one of the current study limitations and needs further investigation.

HCC is the fifth cause of death worldwide and is mostly inducted by the chronic liver disorders, such as viral hepatitis^170,171^. In LC patients, the Wnt signaling was the top research interest and the CTNNB1 was the most assessed gene. The CTNNB1 mutation was also investigated in HCC patients in different studies^118,129,131^. Purcell et al.^73^ reported CTNNB1 mutations in 15% of hepatoblastoma patients while the reported prevalence in Ueda’s study was 75%^74^. Our study subgroup analysis for liver cancer^145^ studies showed that beta-catenin has higher mutation rate (20% (95% CI, 10–31%)) and the most altered pathway was Wnt (17% (95% CI, 11–23%)). It has been indicated that the CTNNB1 and P53 genes are the most involved genes in the HCC^172,173^. Moreover, the conducted studies showed that the P53 mutations were mostly associated with the Asian and African countries, while the CTNNB1 mutations were mostly associated with HCC in the Western countries^172,173^.

The pancreatic cancer is known as the forth cause of cancer mortality in the US with only 10% of the cases living more than 5 years^174^. Witkiewicz et al.^130^ assessed different genes in MAPK/ERK, PI3K/AKT, and Wnt/beta-catenin signaling pathways in pancreatic ductal adenocarcinoma patients. They showed that the AXIN1, KRAS, and PI3CA mutations rate were 5%, 92%, and 4%, respectively. Moreover, the high rate of KRAS mutations in pancreatic cancer patients was confirmed by the other studies^55,119,175^. Our study showed that in the subgroup analysis for pancreatic cancer, the KRAS was the most mutated gene (58% (95% CI: 31–83%)) and MAPK was the most altered pathway (31% (95% CI: 5–66%)). In GC, mutations were 14% (95% CI: 2–34%) for KRAS, 7% (95% CI: 1–17%) for MAPK, and 6% (95% CI: 2–12%) for PI3 pathways. In the pancreatic and gastric cancers, the most evaluated pathways were PI3 and MAPK. The KRAS gene generates a GTPase protein which is critical in regulating the cell proliferation and metabolism^176^. The mutations in KRAS leads to impaired cells activity enhancement and malignancy progression^177^.

Gastric Cancer (GC), as another invasive GI cancer, has significant mortality rate worldwide^178^. Zhang et al.^104^ studied 100 advanced primary GC cases for the purpose of evaluating PI3K/AKT signaling pathway mutations. They suggested that the MAPK/ERK and PI3K/AKT pathways could be potential therapeutic targets for GC treatment^179,180^. The AKT gene produced a protein in the PI3K/Akt pathway which could play a role in tumorogenesis^80^. The mutations in the PIK3CA and AKT in PI3K/AKT pathway could affect downstream signaling pathway genes, like mTOR (mechanistic target of rapamycin kinase) and caspase 9, which are important in GC progression^104,181,182^. Wang et al.^99^ investigated hedgehog pathway in GC patients and showed that the PTCH1 (patched 1) and SMO (smoothened) genes were mutated in 51.2% and 25.6% of the cases. Alterations in PTCH1 gene were associated with the basal cell carcinoma and basal cell nevus syndrome^183,184^.

Moreover, most of the studies included used PCR followed by the Sanger sequencing, as the method of choice. However, some studies used SSCP-PCR (single-strand conformational polymorphism PCR) to detect mutation. The method used the least was the NGS (next generation sequencing) as a preferred method in the recent years. The NGS can be used to analyze numerous samples at the same time and thus reduce the cost and the time required^185^. But the Sanger sequencing is an accurate and sensitive method for mutation analysis and it has been suggested for the confirmation of the NGS results^186^. Also, in the subgroup analysis for the GC, the method of detection could be mentioned as a potent source of the heterogeneity in the current study (Table 3).

The major limitation in the current study was the extent of subject; it is suggested that further investigations use more narrowing strategies. Also, we aimed at minimizing the author bias in data extraction and screening biases using different authors and double check strategies. Also, it should be mentioned that the p53 signaling is not a canonical signaling pathway but due to the p53 non-transcriptional functions, the importance of this pathway in cancer generation, and its interaction with other signaling pathways, in the present study, we assessed p53 as individual pathway^3^.

In conclusion, progression of GI cancers is affected by signaling pathway mediators. Different studies have shown diverse results based on their population, method, and target gene. Our study concluded that the most important genes that are under mutation pressure include KRAS and PI3CA in the CRC, PC, and GC while beta-catenin and CTNNB1 are genes under mutation pressure for liver malignancies. Subgroup analysis and heterogeneity of the studies could illustrate more valid data between different studies for screening strategies. In this regard, signal transduction pathway mutations pooled prevalence was higher in PC and lower in GC (20% vs. 5%). Thus, PC is the most common cancer involved by signal transduction mediator’s mutations. Among studied genes, KRAS in GC and pancreatic cancer and APC in CRC had the most association with cancer outcome. Moreover, MAPK had higher mutation rate among the studied pathways. Furthermore, PCR-SS method had the highest popularity among different methods. Future studies should be carried out to focus on cancer progression and patient’s survival assessments.

## Methods

### Search strategy

In the present comprehensive study, we assessed all relevant original research studies via the electronic literature search in Web of Science (SCIE), PubMed (Including MEDLINE), Science Direct, Scopus, EMBASE, and Google Scholar databases using the keywords including Polymorphism, Mutation, Mutation Rate, Mutation Prevalence, Silent Mutation, Point Mutation, Missense Mutation, INDEL Mutation, Frameshift Mutation, Synonymous Mutation, Non-synonymous Mutation, Transversion Mutation, Transition Mutation, Insertion Mutation, Deletion Mutation, Digestive System Diseases, Gastrointestinal Neoplasms, Digestive System Abnormalities, Biliary Tract Diseases, Biliary Tract Neoplasms, Gallbladder Diseases, Anorectal Malformations, Colorectal Neoplasms, Pancreatic Neoplasms, Hepatocellular carcinoma, Esophageal cancer, Intestinal Diseases, Stomach Diseases, Stomach cancer, Gastric cancer, Liver Diseases, Liver Neoplasms, Pancreatic Diseases, Signaling Pathways, Signal Transduction, Wnt Signaling Pathway, and MAP Kinase Signaling System between January 1998 and September 28, 2019. Also, the reference lists of the screened studies were reviewed so as to find relevant studies (the exact search strategy is available in the supplement data of supplementary Table 1).

### Inclusion and exclusion criteria

The studies were screened by two independent authors and all the studies meeting the inclusion criteria were included. Any discrepancy between the two reviewer authors were sorted out by a third expert. Inclusion criteria were the English language writing, publication up to the date of the search, the study setting of cross-sectional or cohort, and the data eligibility for the study. Furthermore, the meta-analysis, conference seminars, and review articles were excluded from the search results.

### Data extraction

Selected studies were listed in EndNote software (EndNote X7, Thomson Reuters) and were reviewed by two authors of the study independently; disagreements between them were settled by a third expert. All the relevant studies were screened considering the inclusion criteria and the data were extracted. The extracted data included the first author’s name, the publication date (based on year), country, design of the study, type of the cancer, sample size, mutation pathway, gene name, mean age, gender, mutation positive population, and method of detection.

### Risk bias assessment

The risk bias for the non-randomized controlled trials (RCT) was assessed making use of the 13 items in the Research Triangle Institute (RTI), Evidence-based Practice Center^187^.

### Meta-analysis

In this study, to compute of the pooled estimate of prevalence we used the Metaprop command and random models with confidence interval of CI = 95%. The prevalence estimation performed by random effects models in some analyses due to statistically significant of the heterogeneity test. In the present study, for the evaluation of statistical heterogeneity between studies we used Cochran’s Q test and I^2^ statistics. In addition, for the assessment of the source of heterogeneity among studies we used subgroup analysis. Also, funnel plot and Egger test used for the publication bias assessment. For the statistical analysis in this study STATA 16.0 (Stata Corp, College Station, TX, USA) were used by setting the statistical significant value at *p* < 0.05.

## Supplementary information

Supplementary Table 1.

Supplementary Table 2.

Supplementary Table 3.

Supplementary Table 4.

Supplementary Table 5.

Supplementary Table 6.

## Abbreviations

ARMS-PCR: Amplification refractory mutation system polymerase chain reaction
APC: Adenomatous polyposis coli
ARID2: AT-rich interactive domain
ACVR2A: Activin A receptor type 2A
ADAMTS17: A disintegrin-like and metalloprotease
BCLAF1: BCL2 associated transcription factor 1
BTN3A2: Butyrophilin subfamily 3 member A2
BOK: Bcl-2 related ovarian killer
CRC: Colorectal cancer
CDKN2A: Cyclin-dependent kinase inhibitor 2A
CCND1: Cyclin D1
CDHR1: Cadherin related family member 1
CTNNB1: Catenin beta 1
CGH: Comparative genomic hybridization
CISH: Chromogenic in situ hybridization
CAPRIN2: Caprin family member 2
DPC4: Deleted in pancreatic cancer-4
DHPLC: Denaturing high pressure liquid chromatography
ESCC: Esophageal squamous cell carcinoma
EGFR: Epidermal growth factor receptor
FLT3: FMS-like tyrosine kinase 3
FBXW7: F-box and WD repeat domain containing 7
FLG: Human filaggrin gene
GC: Gastric cancer
GI: Gastrointestinal
GBC: Gallbladder carcinoma
GNAS: Guanine nucleotide binding protein, alpha stimulating
CGH: Comparative genomic hybridization
GLTSCR1: Glioma tumor suppressor candidate region gene 1
HBV: Hepatitis B virus
HCV: Hepatitis C virus
HCC: Hepatocellular carcinoma
HPLC: High-performance liquid chromatography
HRM: High resolution melt
IHC: Immunohistochemistry
ISH: In situ hybridization
IPMN/IPMNC: Intraductal papillary mucinous neoplasm/carcinoma
ICC: Intrahepatic cholangiocarcinoma
IGF2R: Insulin like growth factor 2 receptor
JAK1: Janus kinase 1
KDR: Kinase insert domain receptor
KLHL22: Kelch like family member 22
LOH: Loss of heterozygosit
LM: Liver malignancy
mCRC: Metastatic colorectal cancer
MAPK: Mitogen-activated protein kinase
MSS: Microsatellite stable
MSI: Microsatellite instability
mTOR: Mechanistic target of rapamycin kinase
MSI-L: Microsatellite instability low
MSI-H: Microsatellite instability high
NGS: Next-generation sequencing
NOTCH1: Notch receptor 1
PC: Pancreatic cancer
PCR-SS: Polymerase chain reaction-sanger sequencing
PCR-SSCP: Single strand conformation polymorphism polymerase chain reaction
PCR-RFLP: Restriction fragment length polymorphism
PTEN: Phosphatase and tensin homolog
PTP: Protein tyrosine phosphatase
PTPN11: Protein tyrosine phosphatase non-receptor Type 11
PI3K: Phosphatidylinositol 3-kinase
PDGFRA: Platelet derived growth factor receptor alpha
PIK3CA: Phosphatidylinositol-4, 5-bisphosphate 3-kinase catalytic subunit alpha
PMN: Papillary mucinous neoplasm
qRTPCR: Quantitative real-time polymerase chain reaction
RHOA: Ras homolog family member A
RNF169: Ring finger protein 169
RUNX1: Runt-related transcription factor 1
STK11: Serine/threonine kinase 11
SMO: Smoothened, frizzled class receptor
SOX9: SRY-box transcription factor 9
SMAD2: SMAD family member 2
SSCA: Single strand confirmation analysis
SSA/Ps: Sessile serrated adenoma/polyps
SNP: Single-nucleotide polymorphism
TGF-B: Transforming growth factor beta
TRPC4AP: Transient receptor potential cation channel subfamily C member 4 associated protein
VHL: Von Hippel–Lindau
VCAM1: Vascular cell adhesion molecule 1
WISP3: Wntl-inducible signaling pathway protein 3
WGS: Whole genome sequencing
WES: Whole-exome sequencing
