# Supplementary material for: Signal transduction pathway mutations in gastrointestinal (GI) cancers: a systematic review and meta-analysis
Source: Sci Rep. 2020 Oct 30;10:18713. doi: 10.1038/s41598-020-73770-1 (PMC7599243; doi:10.1038/s41598-020-73770-1)
Supplement: Supplementary file 1 — Supplementary Table 1. [file 41598_2020_73770_MOESM1_ESM.docx]

**Supplementary table 1. PubMed Search Strategy using in this study**

| PubMed Search Strategy | ("polymorphism"[Mesh] OR "Mutation"[Mesh] OR "Mutation Rate"[Mesh] OR "Mutation Prevalence"[Mesh] OR "Silent Mutation"[Mesh] OR "Point Mutation"[Mesh] OR "Missense Mutation"[Mesh] OR "INDEL Mutation"[Mesh] OR "Frameshift Mutation"[Mesh] OR "Synonymous Mutation"[Mesh] OR "Non-synonymous Mutation"[Mesh] OR "Transversion Mutation"[Mesh] OR "Transition Mutation"[Mesh] OR "Insertion Mutation"[Mesh] OR "Deletion Mutation"[Mesh] OR "Polymorphism"[tiab] OR "Mutation"[tiab] OR "Mutation Rate"[tiab] OR "Mutation Prevalence"[tiab] OR "Silent Mutation"[tiab] OR "Point Mutation"[tiab] OR "Missense Mutation"[tiab] OR "INDEL Mutation"[tiab] OR "Frameshift Mutation"[tiab] OR "Synonymous Mutation"[tiab] OR "Non-synonymous Mutation"[tiab] OR "Transversion Mutation"[tiab] OR "Transition Mutation"[tiab] OR "Insertion Mutation"[tiab] OR "Deletion Mutation"[tiab]) AND ("Digestive System Diseases"[tiab] OR "Gastrointestinal Neoplasms"[tiab] OR "Digestive System Abnormalities"[tiab] OR "Biliary Tract Diseases"[tiab] OR "Biliary Tract Neoplasms"[tiab] OR "Gallbladder Diseases"[tiab] OR "Anorectal Malformations"[tiab] OR "Colorectal Neoplasms "[tiab] OR "Gastrointestinal Diseases"[tiab] OR "Esophageal Diseases"[tiab] OR "Esophageal cancer"[tiab] OR "Intestinal Diseases"[tiab] OR "Stomach Diseases"[tiab] OR "Stomach cancer"[tiab] OR "Gastric cancer"[tiab] OR "Liver Diseases"[tiab] OR "Liver Neoplasms"[tiab] OR "Pancreatic Diseases"[tiab] OR "Pancreatic Neoplasms"[tiab] OR "Hepatocellular carcinoma"[tiab] OR "Digestive System Diseases"[Mesh] OR "Gastrointestinal Neoplasms"[Mesh] OR "Digestive System Abnormalities"[Mesh] OR "Biliary Tract Diseases"[Mesh] OR "Biliary Tract Neoplasms"[Mesh] OR "Gallbladder Diseases"[Mesh] OR "Anorectal Malformations"[Mesh] OR "Colorectal Neoplasms "[Mesh] OR "Gastrointestinal Diseases"[Mesh] OR "Esophageal Diseases"[Mesh] OR "Esophageal cancer"[Mesh] OR "Intestinal Diseases"[Mesh] OR "Stomach Diseases"[Mesh] OR "Stomach cancer"[Mesh] OR "Gastric cancer"[Mesh] OR "Liver Diseases"[Mesh] OR "Liver Neoplasms"[Mesh] OR "Pancreatic Diseases"[Mesh] OR "Pancreatic Neoplasms"[Mesh] OR "Hepatocellular carcinoma"[Mesh]) AND ("Signaling Pathways"[Mesh] OR "Signal Transduction"[Mesh] OR "Wnt Signaling Pathway"[Mesh] OR "MAP Kinase Signaling System"[Mesh] OR "Signaling Pathways"[tiab] OR "Signal Transduction"[tiab] OR "Wnt Signaling Pathway"[tiab] OR "MAP Kinase Signaling System"[tiab]) |
| --- | --- |
