# Supplementary material for: Signal transduction pathway mutations in gastrointestinal (GI) cancers: a systematic review and meta-analysis
Source: Sci Rep. 2020 Oct 30;10:18713. doi: 10.1038/s41598-020-73770-1 (PMC7599243; doi:10.1038/s41598-020-73770-1)
Supplement: Supplementary file 2 — Supplementary Table 2. [file 41598_2020_73770_MOESM2_ESM.docx]

**krasSupplementary table 2. Gastric cancer (GC) signaling pathway mutation studies analysis (n=16)**

| **No** | **First Author** | **Year** | **Country** | **Population** | | | | **Mutation Analysis** | | | | **Clinic-pathological** | **survival rate** | **Method of detection** | **Ref.** |
| --- | --- | --- | --- | --- | --- | --- | --- | --- | --- | --- | --- | --- | --- | --- | --- |
|  |  |  |  | **Sample**  **Size** | **Mean Age (y) ± SD (Range)** | **Male**  **N (%)** | **Female**  **N (%)** | **Pathway** | **Gene name** | **Exon name** | **Mutation Positive Population %** |  |  |  |  |
| 1 | Shitara | 1999 | Japan | 35 | - | - | - | Smad (TGF-β) | Smad 2 | 1-11 | 0 | - | - | PCR-SSCP, PCR-SS | (1) |
| 2 | Yoo | 2002 | South Korea | 104 | - | - | - | MAPK | HRAS | 1 | 14 | Correlation not identified | - | PCR-SS | (2) |
|  |  |  |  |  |  |  |  |  | KRAS |  |  |  |  |  |  |
| 3 | Clements | 2002 | USA | 73 | - | - | - | Wnt | beta-catenin | 3 | 26 | Correlation not identified | - | PCR-SS | (3) |
| 4 | Li | 2005 | China | 94 | - | - | - | PI3K | PIK3CA | 9-20 | 4.3 | - | - | PCR-SS | (4) |
| 5 | Pan | 2008 | China | 70 | - | 41 | 29 | Wnt | AXIN1 | 7 | 7.1 | - | - | PCR-SS , HPLC | (5) |
|  |  |  |  |  |  |  |  |  | AXIN2 | - |  |  |  |  |  |
|  |  |  |  |  |  |  |  |  | CTNNB1 | 3 |  |  |  |  |  |
| 6 | Kim | 2009 | South Korea | 92 | - | - | - | Wnt | AXIN2 | 8 | 9.8 | Correlation not identified | - | PCR-SSCP | (6) |
|  |  |  |  |  |  |  |  |  | TCF7L2 | 14 | 18.8 |  |  |  |  |
| 8 | Sukawa, | 2012 | Japan | 231 | 71(25-91) | 157 | 74 | PI3 | PIK3CA | 1, 9, 20 | 8.7 | Correlation not identified | - | IHC, PCR-SS | (7) |
| 9 | Lee | 2012 | Korea | 237 | 54.6 | 168 | 69 | Wnt, MAPK, PI3, P53 signaling | PIK3CA | - | 5.1 | - | - | Array | (8) |
|  |  |  |  |  |  |  |  |  | p53 |  | 4.6 |  |  |  |  |
|  |  |  |  |  |  |  |  |  | APC |  | 2.5 |  |  |  |  |
|  |  |  |  |  |  |  |  |  | STK11 |  | 2.1 |  |  |  |  |
|  |  |  |  |  |  |  |  |  | CTNNB1 |  | 1.7 |  |  |  |  |
|  |  |  |  |  |  |  |  |  | CDKN2A |  | 0.8 |  |  |  |  |
| 10 | Hidaka | 2013 | Japan | 130 CGA | 67.2/69.0 ± 9.6 | 107 | 23 | Wnt | CTNNB1 | - | 3 | - | - | PCR-SS | (9) |
|  |  |  |  |  |  |  |  |  | AXIN1 |  | 3.8 |  |  |  |  |
|  |  |  |  |  |  |  |  |  | AXIN2 |  | 4.6 |  |  |  |  |
| 11 | Wang | 2013 | USA | 39 |  |  | - | Hedgehog signaling | PTCH1 | 23 | 51.2 | - | - | PCR-SS | (10) |
|  |  |  |  |  |  |  |  |  | SMO | - | 25.6 |  |  |  |  |
| 12 | Van Grieken | 2013 | UK, Japan, Singapore | 712 | - | - | - | MAPK | KRAS | - | 4.2 | KRAS mutations were less frequent in tumors with lymph node invasion and more frequent in patients >70 years | - | PCR-SS | (11) |
|  |  |  |  |  |  |  |  |  | BRAF |  | 0.14 |  |  |  |  |
| 13 | Zhang | 2014 | China | 100 advanced primary | 60.1 ± 10.1 | 76 | 24 | PI3 | PIK3CA | 18 | 3 | - | - | PCR-SS, HPLC | (12) |
|  |  |  |  |  |  |  |  |  | AKT2 | 6 | 2 |  |  |  |  |
| 14 | Wang | 2014 | USA, China | 262 | - | 113 | 167 | RhoA pathway | RHOA | - | 6.8 | - | - | NGS | (13) |
| 15 | Yoda | 2015 | Japan | 55 | - | 34 | 7 | Wnt | CTNNB1 | - | 3.6% | Correlation not identified | - | NGS | (14) |
|  |  |  |  |  |  |  |  | PI3 (AKT) | PIK3CA, PTPN11 |  | 7.27% |  |  |  |  |
|  |  |  |  |  |  |  |  | MAPK | ERBB2, FLT3, KRAS |  | 20% |  |  |  |  |
| 16 | Zaitsu | 2015 | Japan | 221 | 66.2 | 128 | 67 | PI3 | PTEN | - | 20% | LOH of PTEN was associated with vascular involvement | survival rates were lower in patients with loss of  heterozygosity of PTEN | PCR-SS | (15) |
| 17 | Yang | 2018 | China | 34 | 64± 8.7 | 26 | 8 | MAPK | KRAS | 2 | 0 | Correlation not identified | - | ARMS-PCR | (16) |
|  |  |  |  |  |  |  |  |  | NRAS | 2,3,4 | 0 |  |  |  |  |
|  |  |  |  |  |  |  |  |  | BRAF | 15 | 2.9 |  |  |  |  |

References:

1. Shitara Y, Yokozaki H, Yasui W, Takenoshita S, Kuwano H, Nagamachi Y, et al. No mutations of the Smad2 gene in human sporadic gastric carcinomas. Japanese journal of clinical oncology. 1999;29(1):3-7.

2. Yoo J, Park SY, Robinson RA, Kang SJ, Ahn WS, Kang CS. ras Gene mutations and expression of Ras signal transduction mediators in gastric adenocarcinomas. Archives of pathology & laboratory medicine. 2002;126(9):1096-100.

3. Clements WM, Wang J, Sarnaik A, Kim OJ, MacDonald J, Fenoglio-Preiser C, et al. β-catenin mutation is a frequent cause of Wnt pathway activation in gastric cancer. Cancer Research. 2002;62(12):3503-6.

4. Li VSW, Wong CW, Chan TL, Chan ASW, Zhao W, Chu KM, et al. Mutations of PIK3CA in gastric adenocarcinoma. BMC Cancer. 2005;5.

5. Pan KF, Liu WG, Zhang L, You WC, Lu YY. Mutations in components of the Wnt signaling pathway in gastric cancer. World journal of gastroenterology. 2008;14(10):1570-4.

6. Kim MS, Kim SS, Ahn CH, Yoo NJ, Lee SH. Frameshift mutations of Wnt pathway genes AXIN2 and TCF7L2 in gastric carcinomas with high microsatellite instability. Human Pathology. 2009;40(1):58-64.

7. Sukawa Y, Yamamoto H, Nosho K, Kunimoto H, Suzuki H, Adachi Y, et al. Alterations in the human epidermal growth factor receptor 2-phosphatidylinositol 3-kinase-v-Akt pathway in gastric cancer. World journal of gastroenterology. 2012;18(45):6577-86.

8. Lee J, Van Hummelen P, Go C, Palescandolo E, Jang J, Park HY, et al. High-throughput mutation profiling identifies frequent somatic mutations in advanced gastric adenocarcinoma. PloS one. 2012;7(6):e38892.

9. Hidaka Y, Mitomi H, Saito T, Takahashi M, Lee SY, Matsumoto K, et al. Alteration in the Wnt/beta-catenin signaling pathway in gastric neoplasias of fundic gland (chief cell predominant) type. Hum Pathol. 2013;44(11):2438-48.

10. Wang XD, Inzunza H, Chang H, Qi Z, Hu B, Malone D, et al. Mutations in the hedgehog pathway genes SMO and PTCH1 in human gastric tumors. PLoS One. 2013;8(1):e54415.

11. Van Grieken NCT, Aoyma T, Chambers PA, Bottomley D, Ward LC, Inam I, et al. KRAS and BRAF mutations are rare and related to DNA mismatch repair deficiency in gastric cancer from the East and the West: Results from a large international multicentre study. British Journal of Cancer. 2013;108(7):1495-501.

12. Zhang QY, Cheng WX, Li WM, Au W, Lu YY. Occurrence of low frequency PIK3CA and AKT2 mutations in gastric cancer. Mutation research. 2014;769:108-12.

13. Wang K, Yuen ST, Xu J, Lee SP, Yan HHN, Shi ST, et al. Whole-genome sequencing and comprehensive molecular profiling identify new driver mutations in gastric cancer. Nature Genetics. 2014;46(6):573-82.

14. Yoda Y, Takeshima H, Niwa T, Kim JG, Ando T, Kushima R, et al. Integrated analysis of cancer-related pathways affected by genetic and epigenetic alterations in gastric cancer. Gastric cancer : official journal of the International Gastric Cancer Association and the Japanese Gastric Cancer Association. 2015;18(1):65-76.

15. Zaitsu Y, Oki E, Ando K, Ida S, Kimura Y, Saeki H, et al. Loss of heterozygosity of PTEN (encoding phosphate and tensin homolog) associated with elevated HER2 expression is an adverse prognostic indicator in gastric cancer. Oncology. 2015;88(3):189-94.

16. Yang Q, Huo S, Sui Y, Du Z, Zhao H, Liu Y, et al. Mutation status and immunohistochemical correlation of KRAS, NRAS, and BRAF in 260 Chinese colorectal and gastric cancers. Frontiers in Oncology. 2018;8(OCT).
