# Supplementary material for: Signal transduction pathway mutations in gastrointestinal (GI) cancers: a systematic review and meta-analysis
Source: Sci Rep. 2020 Oct 30;10:18713. doi: 10.1038/s41598-020-73770-1 (PMC7599243; doi:10.1038/s41598-020-73770-1)
Supplement: Supplementary file 3 — Supplementary Table 3. [file 41598_2020_73770_MOESM3_ESM.docx]

**Supplementary table 3. Colorectal cancer (CRC) signaling pathway mutation studies analysis (n=65)**

| **No** | **First**  **Author’s** | **Year** | **Country** | **Population** | | | | **Mutation Analysis** | | | **Mutation Positive Population %** | **Clinic-pathological** | **survival rate** | **Method of detection** | **Ref.** |
| --- | --- | --- | --- | --- | --- | --- | --- | --- | --- | --- | --- | --- | --- | --- | --- |
|  |  |  |  | **Sample**  **Size** | **Mean Age (y) ± SD (Range)** | **Male**  **N (%)** |  |  | **Gene name** | **Exon name** |  |  |  |  |  |
| 1 | Müller | 1998 | Germany | 23 | - | **-** | - | Wnt | beta-catenin | - | 4.3 | - | - | PCR-SS | (1) |
| 2 | Sparks | 1998 | USA | 27 | - | **-** | - | Wnt | CTNNB1 | - | 48 | - | - | PCR-SS | (2) |
| 3 | Koyama | 1999 | Japan | 73 | - | **-** | - | Smad (TGF-β) | DPC4/  SMAD4 | 1-11 | 10 | - | - | PCR-SSCP, PCR-SS, LOH | (3) |
| 4 | Mirabelli | 1999 | Canada | 53 | <50 | **-** |  | Wnt | beta-catenin | 3 | 25 | - | - | PCR-SS | (4) |
| 5 | Fujimori | 2001 | Japan | 72 | 55.4 ± 13.7 | 29 | 43 | Wnt | beta-catenin | 3 | 37.5 | Immunohistochemistry of cyclin D1 were more in beta-catenin mutated cases | - | PCR-SS | (5) |
| 6 | Shitoh | 2001 | Japan | 56 | **-** | - | - | Wnt | APC | 15 A, C, D, G, H, I | 50% MSI-H  41% MSS/MSI-L | beta Catenin mutation in exon 3 was higher in MSI-H | - | PCR-SSCP | (6) |
|  |  |  |  |  |  |  |  |  | beta-catenin | 3 | 27% MSI-H  3% MSS/MSI-L |  |  |  |  |
| 7 | Engeland | 2002 | Netherlands | 222 | 67.9 | 129 | 93 | MAPK | KRAS | - | 39 | - | - | PCR, SS | (7) |
| 8 | Yuen | 2002 | UK | 215 | - | 122 | 93 | MAPK | BRAF | - | 5.1 | adenomas with KRAS mutation were larger/ / KRAS mutations were more often in  villous or tubulovillous morphology | - | PCR, SS | (8) |
| 9 | Jass | 2003 | Australia | 95 | - | - | - | Wnt | APC | 14 | 28.4 | APC mutation was  lower in sporadic MSI-H | - | PCR, DHPLC | (9) |
| 10 | Zhang | 2003 | Japan | 74 | 68.3 (33-39) | 41 | 33 | Wnt and MAPK | beta-catenin | - | 27% with nuclear accumulation  26% with tumor invasion front | - | - | PCR, SS | (10) |
|  |  |  |  |  |  |  |  |  | KRAS | - | 42 |  |  |  |  |
| 11 | Fransén | 2004 | Sweden | 130 |  | 60 | 68 | MAPK | BRAF | - | 11.5 | tumors without BRAF and KRAS mutations  displaying MSI/ BRAF mutated tumors were located in the colon | - | PCR-SS, SSCA | (11) |
|  |  |  |  |  |  |  |  |  | KRAS |  | 40 |  |  |  |  |
| 12 | Sakamoto | 2004 | Japan | 48 | 63.7(44-88) | 42 | 6 | MAPK | KRAS | 1, 2 | 14.6 | tumors without KRAS mutations were Ras association domain family 1 gene (RASSF1) methylation | - | PCR-SS | (12) |
| 13 | Pasche | 2005 | USA | 44 | - | - | - | Smad (TGF-β) | TGF-β | 1 | 29.5 | - | - | PCR, CGH, LOH | (13) |
| 14 | Thorstensen | 2005 | Norway | 310 | 68.3 (24– 92) | 158 | 151 | Wnt | AXIN2 | 7 | 20 | APC and p53 mutations were more in MSS/ p53 and chromosome instability were associated | - | PCR-SS | (14) |
|  |  |  |  |  |  |  |  |  | PTEN | 8 | 17 |  |  |  |  |
|  |  |  |  |  |  |  |  |  | TCF4 | 17 | 46 |  |  |  |  |
|  |  |  |  |  |  |  |  |  | WISP3 | 4 | 28 |  |  |  |  |
|  |  |  |  |  |  |  |  |  | p53 | - | 18 |  |  |  |  |
| 15 | Mikami | 2006 | Japan | 310 | 66.0 ± 10.4 | 198 | 112 | Wnt, MAPK, PI3 | BRAF | - | 5.4 | beta-catenin mutation  was higher in flat-type tumors with depressed areas/ KRAS mutation was correlated  with age, size and gender | - | PCR-SS | (15) |
|  |  |  |  |  |  |  |  |  | KRAS | - | 21.6 |  |  |  |  |
|  |  |  |  |  |  |  |  |  | PIK3C | 9-20 | 1 |  |  |  |  |
|  |  |  |  |  |  |  |  |  | beta-catenin | 3 | 7.1 |  |  |  |  |
| 16 | Noda | 2006 | Japan | 73 | - | - | - | MAPK | BRAF | - | 4.1 | - | - | - | (16) |
|  |  |  |  |  |  |  |  |  | KRAS | - | 16.4 |  |  |  |  |
| 17 | Ching-Shian Leong | 2008 | Malaysia | 24 | - | - | - | PI3 | PIK3C | 9, 20 | 0 | - | - | PCR-SS | (17) |
| 18 | Seth | 2009 | UK | 37 | - | **-** | - | MAPK | KRAS | 2, 3, 4 | 49 | BRAF mutations were associated with CpG island methylation | - | PCR-SS | (18) |
|  |  |  |  |  |  |  |  |  | BRAF | 11, 13, 14, 15 | 10 |  |  |  |  |
| 19 | Kim | 2009 | South Korea | 71 | - | - | - | Wnt | AXIN2 | 8 | 1.4 | - | - | PCR-SSCP | (19) |
|  |  |  |  |  |  |  |  |  | TCF7L2 | 14 | 5.6 |  |  |  |  |
| 20 | Packham | 2009 | Australia | 1198 | - | - | - | MAPK | KRAS | - | 32.4 | - | - | Real-time PCR, PCR, pyro sequencing | (20) |
|  |  |  |  |  |  |  |  |  | BRAF |  | 13.2 |  |  |  |  |
| 21 | Baldus | 2010 | Germany | 100 | 65 (18-94) | 56 | 44 | MAPK, PI3 | KRAS | 2 | 41 | KRAS mutations correlated with lymph node metastases / BRAF mutations were in poorly differentiated | - | PCR, pyro sequencing | (21) |
|  |  |  |  |  |  |  |  |  | BRAF | 15 | 7 |  |  |  |  |
|  |  |  |  |  |  |  |  |  | PIK3CA | 9, 20 | 21 |  |  |  |  |
| 22 | Irahara | 2010 | USA | 225 | 67.4 ± 8.7 | 108 | 117 | MAPK, PI3 | NRAS | - | 2.2 | - | - | PCR, pyro sequencing | (22) |
|  |  |  |  |  |  |  |  |  | KRAS |  | 41 |  |  |  |  |
|  |  |  |  |  |  |  |  |  | BRAF |  | 14 |  |  |  |  |
|  |  |  |  |  |  |  |  |  | PIK3CA |  | 11 |  |  |  |  |
| 23 | Smith | 2010 | UK | 106 | 67(45-80) | 64 | 42 | MAPK | KRAS | - | 9.4 | - | - | PCR-RFLP | (23) |
| 24 | Liao | 2010 | China | 61 | **-** | 43 | 18 | MAPK, PI3 | KRAS | 12, 13 | 19.7 |  | Not correlated | PCR-SS | (24) |
|  |  |  |  |  |  |  |  |  | BRAF | V600 | 4.9 |  |  |  |  |
|  |  |  |  |  |  |  |  |  | PIK3CA | 9, 20 | 4.9 |  |  |  |  |
|  |  |  |  |  |  |  |  |  | EGFR | 19, 21 | 0 |  |  |  |  |
| 25 | Sameer | 2011 | India | 86 | - | 49 | 37 | Wnt | SMAD4 | 2, 9, 10, 11 | 18.6 | SMAD4 mutations were more in colon tumors, node involvement, grade C and D and with KRAS mutations | - | PCR-SSCP, PCR, SS | (25) |
|  |  |  |  |  |  |  |  |  | KRAS | 1 | 24 |  |  |  |  |
| 26 | Watanabe | 2011 | Japan | 113 | 66 (26-87) | 76 | 37 | MAPK | KRAS | - | 31 | - | - | Real-time PCR | (26) |
| 27 | Metzger | 2011 | Belgium | 236 | - | - | - | MAPK | EGFR | 18 | 2.1 | - | - | PCR-SS | (27) |
|  |  |  |  |  |  |  |  |  |  | 20 | 0.4 |  |  |  |  |
| 28 | Naghibalhossaini | 2011 | Iran | 110 | - | 72 | 38 | MAPK | KRAS | - | 28 | KRAS mutations were more in tumors with left location | - | PCR-RFLP | (28) |
|  |  |  |  |  |  |  |  |  | BRAF |  | 0 |  |  |  |  |
| 29 | Khiari | 2012 | Tunisia | 150 | - | - | - | Wnt | CTNNB1 | 3 | 1.3 | exon 3 deletion were associated with tumor invasion |  | PCR | (29) |
| 30 | Tai | 2012 | Taiwan | 126 | 64 (28-93) | 75 | 51 | MAPK | KRAS | - | 32.5 | Tumor invasion and stage were associated with KRAS mutation | - | PCR-SS | (30) |
| 31 | Bond | 2012 | Australia | 1081 | - | - | - | MAPK | BRAF | - | 13.5 | Age, gender and tumor location were associated with BRAF mutations |  | HRM | (31) |
|  |  |  |  |  |  |  |  |  | KRAS |  | 43.6 |  |  |  |  |
|  |  |  |  |  |  |  |  | P53 signaling | p53 |  | 40-46 |  |  |  |  |
| 32 | Laghi | 2012 | Italy | 1041 | - | - | - | MAPK | KRAS | Codon 12, 13 | 20 | - | - | PCR-SS | (32) |
|  |  |  |  |  |  |  |  |  | BRAF | - | 36.7 |  |  |  |  |
| 33 | Levidou | 2012 | Greece | 94 | 60 (35-82) | 53 | 41 | MAPK | KRAS | - | 23.4 | - | - | PCR-SS | (33) |
|  |  |  |  |  |  |  |  |  | BRAF | 15 | 12.7 |  |  |  |  |
|  |  |  |  |  |  |  |  |  | KRAS BRAF | - | 37 |  |  |  |  |
| 34 | Voorham | 2012 | Netherlands | 106 | - | - | - | Wnt, MAPK, Smad (TGF-β) | PIK3CA | - | 0 | flat adenomas and polypoid adenomas were different KRAS mutations in codon 12 | - | high-throughput genotyping, PCR-SS | (34) |
|  |  |  |  |  |  |  |  |  | PIK3R1 |  | 0 |  |  |  |  |
|  |  |  |  |  |  |  |  |  | EGFR |  | 0 |  |  |  |  |
|  |  |  |  |  |  |  |  |  | PTEN |  | 0 |  |  |  |  |
|  |  |  |  |  |  |  |  |  | MAP2K4 |  | 0 |  |  |  |  |
|  |  |  |  |  |  |  |  |  | SMAD4 |  | 0 |  |  |  |  |
|  |  |  |  |  |  |  |  |  | STK11 |  | 0 |  |  |  |  |
|  |  |  |  |  |  |  |  |  | PDGFRA |  | 0 |  |  |  |  |
|  |  |  |  |  |  |  |  |  | KRAS |  | 36.3 |  |  |  |  |
|  |  |  |  |  |  |  |  |  | BRAF |  | 2.83 |  |  |  |  |
|  |  |  |  |  |  |  |  |  | NRAS |  | 0.94 |  |  |  |  |
|  |  |  |  |  |  |  |  |  | FBXW1 |  | 1.13 |  |  |  |  |
|  |  |  |  |  |  |  |  |  | CTNNB1 |  | 0.94 |  |  |  |  |
|  |  |  |  |  |  |  |  |  | APC |  | 30.27 |  |  |  |  |
| 35 | Whitehall | 2012 | Australia | 829 | 67 | 433 | 396 | MAPK, PI3 | KRAS | - | 34.6 | PIK3CA exon 9 mutations associated with tumor location/PIK3CA mutations associated with CpG Island Methylation | - | PCR-SS | (35) |
|  |  |  |  |  |  |  |  |  | BRAF |  | 15.2 |  |  |  |  |
|  |  |  |  |  |  |  |  |  | PIK3CA |  | 14 |  |  |  |  |
| 36 | Gurzu | 2013 | Romania | 170 | 59.58±11.57 | 120 | 50 | MAPK | BRAF | - | 8.23 | Tumors in in the distal colorectal segments associated with MLH1, BRAF, KRAS and MSS | - | PCR-SS | (36) |
|  |  |  |  |  |  |  |  |  | KRAS | codon 12 | 41.7 |  |  |  |  |
| 37 | Fleming | 2013 | USA | 744 | 62.9(25- 99) | 414 | 330 | Smad (TGF-β) | SMAD4 | - | 8.6 | SMAD4 associated with Mucinous tumor feature, SMAD3 associated with tumor differentiation and gender; SMAD2 associated with chromosomal instability | - | PCR-SS | (37) |
|  |  |  |  |  |  |  |  |  | SMAD2 |  | 3.4 |  |  |  |  |
|  |  |  |  |  |  |  |  |  | SMAD3 |  | 4.3 |  |  |  |  |
| 38 | Aissi | 2013 | Tunisia | 51 | - | 12 | 4 | MAPK | KRAS |  | 31.5 | - | - | PCR-SS | (38) |
| 39 | Han | 2013 | Korea | 60 | 63.5 (35–86) | 40 | 20 | MAPK | KRAS | - | 40 | - | - | NGS | (39) |
|  |  |  |  |  |  |  |  | Wnt | APC |  | 58 |  |  |  |  |
|  |  |  |  |  |  |  |  | - | TTN |  | 35 |  |  |  |  |
|  |  |  |  |  |  |  |  |  | FBXW7 |  | 25 |  |  |  |  |
|  |  |  |  |  |  |  |  |  | SMAD4 |  | 8 |  |  |  |  |
|  |  |  |  |  |  |  |  |  | MAFB |  | 2 |  |  |  |  |
|  |  |  |  |  |  |  |  |  | GNAS |  | 7 |  |  |  |  |
|  |  |  |  |  |  |  |  |  | SRC |  | 0 |  |  |  |  |
|  |  |  |  |  |  |  |  | P53 signaling | p53 |  | 45 |  |  |  |  |
| 40 | Neumann | 2013 | Germany | 171 | - | 92 | 79 | MAPK | KRAS | 2 | 40.9 | BRAF exon 15 were associated with gender, tumor grade and mismatch repair deficiency / KRAS exon were associated with metastasis | - | PCR-SS | (40) |
|  |  |  |  |  |  |  |  |  | BRAF | 15 | 11.1 |  |  |  |  |
|  |  |  |  |  |  |  |  |  | PIK3CA | 9, 20 | 18.7 |  |  |  |  |
|  |  |  |  |  |  |  |  |  | AKT | 3 | 0.6 |  |  |  |  |
| 41 | Shen | 2013 | China | 676 | - | 470 | 269 | MAPK | KRAS | codons 12, 13, 61 | 35.9 | KRAS mutation associated with gender and age / PIK3 mutation associated with tumor location/ NRAS and tumor stage | - | PCR-SS | (41) |
|  |  |  |  |  |  |  |  |  | BRAF | 11, 15 | 6.96 |  |  |  |  |
|  |  |  |  |  |  |  |  |  | PIK3CA | 9, 20 | 9.9 |  |  |  |  |
|  |  |  |  |  |  |  |  |  | NRAS | codons12, 13, 61 | 4.19 |  |  |  |  |
| 42 | Yip | 2013 | Malaysia | 49 | - | 24 | 17 | MAPK, PI3 | KRAS | - | 25.0 | - | - | PCR-SS | (42) |
|  |  |  |  |  |  |  |  |  | BRAF | 15 | 2.3 |  |  |  |  |
|  |  |  |  |  |  |  |  |  | PTEN | 1-9 | 0 |  |  |  |  |
| 43 | Chen | 2013 | Taiwan | 195 | - | 94 | 101 | Wnt | APC | - | 33.8 | - | - | PCR-SS | (43) |
| 44 | Saigusa | 2013 | Japan | 74 | 69 | 125 | 87 | MAPK | KRAS | - | 29.7 | - | - | PCR-SS | (44) |
| 45 | Shi | 2013 | China | 28 | - | - | - | MAPK | BRAF | - | 78.6 | - | - | - | (45) |
| 46 | Zhu | 2014 | China | 148 | - | 90 | 58 | MAPK, PI3 | KRAS |  | 31.1 | KRAS and PIK3CA associated with tumor stage and metastasis | - | PCR, pyro sequencing | (46) |
|  |  |  |  |  |  |  |  |  | BRAF |  | 7.4 |  |  |  |  |
|  |  |  |  |  |  |  |  |  | PIK3CA |  | 9.5 |  |  |  |  |
| 47 | Chang | 2014 | Taiwan | 85 | - | 60 | 25 | MAPK, PI3 | KRAS | - | 18.94 | - | - | PCR-SS | (47) |
|  |  |  |  |  |  |  |  |  | BRAF |  | 3.15 |  |  |  |  |
|  |  |  |  |  |  |  |  |  | PIK3CA |  | 5.2 |  |  |  |  |
| 48 | Ahn | 2014 | Korea | 164 | - | 97 | 67 | MAPK | KRAS | - | 43.3 | correlation was not identified | - | real-time PCR | (48) |
|  |  |  |  |  |  |  |  |  | BRAF |  | 15.9 |  |  |  |  |
| 49 | Tong | 2014 | China | 1506 | 61 ± 11.3 | 889 | 617 | MAPK | KRAS | Codon 12 | 75.1 | KRAS mutations were associated with gender and tumor site | - | PCR-SS | (49) |
|  |  |  |  |  |  |  |  |  |  | Codon 13 | 19.3 |  |  |  |  |
|  |  |  |  |  |  |  |  |  |  | Codon 61 | 2.5 |  |  |  |  |
|  |  |  |  |  |  |  |  |  |  | 146 | 2.7 |  |  |  |  |
| 50 | Mohammadi-asl | 2014 | Iran | 80 | - | 44 | 36 | MAPK | BRAF | 15 | 46.25 | correlation was not identified | - | PCR-SS | (50) |
| 51 | Chen | 2014 | China | 214 | - | - | - | MAPK, PI3 | KRAS | 2 | 44.9 | PIK3CA were associated with tumor location / BRAF exon 15 associated with tumor location, stage and metastasis / KRAS exon 2 associated with tumor stage | KRAS codon 13 mutations are associated  with poor survival | PCR-SS | (51) |
|  |  |  |  |  |  |  |  |  | BRAF | 15 | 4.2 |  |  |  |  |
|  |  |  |  |  |  |  |  |  | PIK3CA | 9, 20 | 12.3 |  |  |  |  |
| 52 | Lee | 2014 | Korea | 53 | 61 (43-81) | - | - | - | APC | - | 73 | - | - | NGS | (52) |
|  |  |  |  |  |  |  |  |  | KRAS | - | 33 |  |  |  |  |
|  |  |  |  |  |  |  |  |  | ARID1A, PIK3CA | - | 6.7 |  |  |  |  |
| 53 | Kawamata | 2015 | Japan | 43 liver metastatic tumors | - | 19 | 24 | MAPK | KRAS | - | 39.5 | correlation was not identified | No correlation | SSCA, SS | (53) |
| 54 | Lan | 2015 | Taiwan | 1492 | - | - | - | MAPK, PI3 | PIK3CA | - | 14.3 | RAS or PI3K mutation were  associated with a proximal location, mucinous histology, and microsatellite instability/ RAS with elevated serum CEA level/ PI3K with lymphovascular invasion | - | MALDI–TOF mass spectrometry | (54) |
|  |  |  |  |  |  |  |  |  | KRAS |  | 47.3 |  |  |  |  |
| 55 | Samara | 2015 | Greek | 332 | 67 ± 10.7 | 180 | 142 | MAPK | KRAS | 2 | 39 | KRAS mutations were associated with location and differentiation | - | PCR-SS | (55) |
|  |  |  |  | 188 |  |  |  |  | BRAF | 15 | 9 |  |  |  |  |
| 56 | Abdelmaksoud | 2015 | Tunisia | 124 | 62.9 | 72 | 52 | Wnt | APC | 15 | 52 | - | - | PCR-SS | (56) |
|  |  |  |  |  |  |  |  |  | CTNNB1 | 3 | 16 |  |  |  |  |
| 57 | Kawazoe | 2015 | Japan | 264 | 64 (32–86) | 166 | 94 | MAPK, PI3 | KRAS | 2 | 34 | BRAF mutations were associated with tumor location, site of metastasis and differentiation | Survival of patients with any of the mutations was shorter | PCR, ISH | (57) |
|  |  |  |  |  |  |  |  |  | KRAS | 3, 4 | 3.8 |  |  |  |  |
|  |  |  |  |  |  |  |  |  | NRAS | - | 4.2 |  |  |  |  |
|  |  |  |  |  |  |  |  |  | BRAF | - | 5.4 |  |  |  |  |
|  |  |  |  |  |  |  |  |  | PIK3CA | - | 6.4 |  |  |  |  |
| 58 | Lin | 2015 | USA | 113  MSS | - | 29 | 113 | Wnt, PI3, MAPK, Smad, mTOR | APC | - | 47 | MSS tumors with APC mutations were more frequent |  | NGS | (58) |
|  |  |  |  |  |  |  |  |  | BRAF |  | 5 |  |  |  |  |
|  |  |  |  |  |  |  |  |  | CTNNB1 |  | 5 |  |  |  |  |
|  |  |  |  |  |  |  |  |  | KRAS |  | 46 |  |  |  |  |
|  |  |  |  |  |  |  |  |  | NRAS |  | 4 |  |  |  |  |
|  |  |  |  |  |  |  |  |  | PIK3CA |  | 15 |  |  |  |  |
|  |  |  |  |  |  |  |  |  | SMAD4 |  | 11 |  |  |  |  |
|  |  |  |  |  |  |  |  |  | p53 |  | 63 |  |  |  |  |
|  |  |  |  |  |  |  |  |  | AKT1 |  | 1 |  |  |  |  |
|  |  |  |  |  |  |  |  |  | PTEN |  | 3 |  |  |  |  |
|  |  |  |  |  |  |  |  |  | mTOR |  | 18 |  |  |  |  |
|  |  |  |  | 29  MSI |  |  |  |  | APC | - | 34 |  |  |  |  |
|  |  |  |  |  |  |  |  |  | BRAF |  | 34 |  |  |  |  |
|  |  |  |  |  |  |  |  |  | CTNNB1 |  | 3 |  |  |  |  |
|  |  |  |  |  |  |  |  |  | KRAS |  | 31 |  |  |  |  |
|  |  |  |  |  |  |  |  |  | NRAS |  | 10 |  |  |  |  |
|  |  |  |  |  |  |  |  |  | PIK3CA |  | 34 |  |  |  |  |
|  |  |  |  |  |  |  |  |  | SMAD4 |  | 0 |  |  |  |  |
|  |  |  |  |  |  |  |  |  | p53 |  | 31 |  |  |  |  |
|  |  |  |  |  |  |  |  |  | AKT1 |  | 3 |  |  |  |  |
|  |  |  |  |  |  |  |  |  | PTEN |  | 28 |  |  |  |  |
|  |  |  |  |  |  |  |  |  | mTOR |  | 59 |  |  |  |  |
| 59 | Grellety | 2016 | France | 1791 | 72(89-28) | - | - | MAPK | KRAS | - | 31.1 | - | - | NGS | (59) |
|  |  |  |  |  |  |  |  |  | BRAF | - | 26.6 |  |  |  |  |
|  |  |  |  |  |  |  |  |  | NRAS | - | 35.3 |  |  |  |  |
| 60 | Dallol | 2016 | Saudi Arabia | 99 | - | 58 | 41 | Wnt, PI3, MAPK, P53 signaling | p53 | - | 65 |  | EGFR mutations were associated with survival | NGS | (60) |
|  |  |  |  |  |  |  |  |  | APC |  | 36 |  |  |  |  |
|  |  |  |  |  |  |  |  |  | KRAS |  | 35 |  |  |  |  |
|  |  |  |  |  |  |  |  |  | PIK3CA |  | 19 |  |  |  |  |
|  |  |  |  |  |  |  |  |  | PTEN |  | 13 |  |  |  |  |
|  |  |  |  |  |  |  |  |  | EGFR |  | 11 |  |  |  |  |
|  |  |  |  |  |  |  |  |  | SMAD4 |  | 11 |  |  |  |  |
|  |  |  |  |  |  |  |  |  | FBXW7 |  | 7 |  |  |  |  |
| 61 | Jauhri | 2016 | India | 112 | - | - | - | - | KDR | - | 19.6 | - | - | NGS | (61) |
|  |  |  |  |  |  |  |  |  | PTEN | - | 17 |  |  |  |  |
|  |  |  |  |  |  |  |  |  | FBXW7 | - | 10.7 |  |  |  |  |
|  |  |  |  |  |  |  |  |  | SMAD4 | - | 8 |  |  |  |  |
|  |  |  |  |  |  |  |  |  | VHL | - | 10.7 |  |  |  |  |
|  |  |  |  |  |  |  |  |  | KIT | - | 8 |  |  |  |  |
|  |  |  |  |  |  |  |  |  | MET | - | 7.1 |  |  |  |  |
|  |  |  |  |  |  |  |  |  | ATM | - | 6.3 |  |  |  |  |
|  |  |  |  |  |  |  |  |  | CTNNB1 | - | 4.5 |  |  |  |  |
|  |  |  |  |  |  |  |  |  | CDKN2A | - | 4.5 |  |  |  |  |
| 62 | Nam | 2016 | Korea | 191 | 60(28-93) | 103 | 88 | MAPK, PI3 | KRAS | - | 54.5 | correlation was not identified | BRAF mutations were associated with the worst prognosis | Real-time PCR, PCR-SS | (62) |
|  |  |  |  |  |  |  |  |  | BRAF |  | 3.1 |  |  |  |  |
|  |  |  |  |  |  |  |  |  | PIK3CA |  | 13 |  |  |  |  |
| 63 | Ziv | 2017 | USA | 40 | 60 (33-82) | - | - | MAPK | MAPK | - | 45 | - | PI3K pathway were associated with survival | - | (63) |
|  |  |  |  |  |  |  |  |  | PI3K | 1,9, 20 | 25 |  |  |  |  |
| 64 | Mizuno | 2018 | USA | 278 mCRC | - | - | - | Smad (TGF-β) | SMAD4 | - | 13 | SMAD4 mutation associated with lymph node metastases | SMAD4 and RAS mutations shows worse survival | PCR-SS | (64) |
| 65 | Yang | 2018 | China | 86 | 63± 11 | 51 | 35 | MAPK | KRAS | 2 | 42.12 | KRAS mutation associated with tumor location, type, differentiation and gender | - | ARMS-PCR | (65) |
|  |  |  |  |  |  |  |  |  | NRAS | 2,3,4 | 1.2 |  |  |  |  |
|  |  |  |  |  |  |  |  |  | BRAF | 15 | 3.5 |  |  |  |  |

**References:**

1. Müller O, Nimmrich I, Finke U, Friedl W, Hoffmann I. A β-catenin mutation in a sporadic colorectal tumor of the RER phenotype and absence of β-catenin germline mutations in FAP patients. Genes Chromosomes and Cancer. 1998;22(1):37-41.

2. Sparks AB, Morin PJ, Vogelstein B, Kinzler KW. Mutational analysis of the APC/β-catenin/Tcf pathway in colorectal cancer. Cancer Research. 1998;58(6):1130-4.

3. Koyama M, Ito M, Nagai H, Emi M, Moriyama Y. Inactivation of both alleles of the DPC4/SMAD4 gene in advanced colorectal cancers: Identification of seven novel somatic mutations in tumors from Japanese patients. Mutation Research - Mutation Research Genomics. 1999;406(2-4):71-7.

4. Mirabelli-Primdahl L, Gryfe R, Kim H, Millar A, Luceri C, Dale D, et al. Beta-catenin mutations are specific for colorectal carcinomas with microsatellite instability but occur in endometrial carcinomas irrespective of mutator pathway. Cancer Res. 1999;59(14):3346-51.

5. Fujimori M, Ikeda S, Shimizu Y, Okajima M, Asahara T. Accumulation of beta-catenin protein and mutations in exon 3 of beta-catenin gene in gastrointestinal carcinoid tumor. Cancer Res. 2001;61(18):6656-9.

6. Shitoh K, Furukawa T, Kojima M, Konishi F, Miyaki M, Tsukamoto T, et al. Frequent activation of the beta-catenin-Tcf signaling pathway in nonfamilial colorectal carcinomas with microsatellite instability. Genes, chromosomes & cancer. 2001;30(1):32-7.

7. Engeland M, Roemen GMJM, Brink M, Pachen MMM, Weijenberg MP, De Bruïne AP, et al. K-ras mutations and RASSF1A promoter methylation in colorectal cancer. Oncogene. 2002;21(23):3792-5.

8. Yuen ST, Davies H, Chan TL, Ho JW, Bignell GR, Cox C, et al. Similarity of the phenotypic patterns associated with BRAF and KRAS mutations in colorectal neoplasia. Cancer Research. 2002;62(22):6451-5.

9. Jass JR, Barker M, Fraser L, Walsh MD, Whitehall VLJ, Gabrielli B, et al. APC mutation and tumour budding in colorectal cancer. Journal of Clinical Pathology. 2003;56(1):69-73.

10. Zhang B, Ougolkov A, Yamashita K, Takahashi Y, Mai M, Minamoto T. beta-Catenin and ras oncogenes detect most human colorectal cancer. Clinical cancer research : an official journal of the American Association for Cancer Research. 2003;9(8):3073-9.

11. Fransén K, Klintenäs M, Österström A, Dimberg J, Monstein HJ, Söderkvist P. Mutation analysis of the BRAF, ARAF and RAF-1 genes in human colorectal adenocarcinomas. Carcinogenesis. 2004;25(4):527-33.

12. Sakamoto N, Terai T, Ajioka Y, Abe S, Kobayasi O, Hirai S, et al. Frequent hypermethylation of RASSF1A in early flat-type colorectal tumors. Oncogene. 2004;23(55):8900-7.

13. Pasche B, Knobloch TJ, Bian Y, Liu J, Phukan S, Rosman D, et al. Somatic acquisition and signaling of TGFBR1*6A in cancer. Jama. 2005;294(13):1634-46.

14. Thorstensen L, Lind GE, Lovig T, Diep CB, Meling GI, Rognum TO, et al. Genetic and epigenetic changes of components affecting the WNT pathway in colorectal carcinomas stratified by microsatellite instability. Neoplasia (New York, NY). 2005;7(2):99-108.

15. Mikami M, Nosho K, Yamamoto H, Takahashi T, Maehata T, Taniguchi H, et al. Mutational analysis of β-catenin and the RAS-RAF signalling pathway in early flat-type colorectal tumours. European Journal of Cancer. 2006;42(17):3065-72.

16. Noda H, Kato Y, Yoshikawa H, Arai M, Togashi K, Nagai H, et al. Frequent involvement of ras-signalling pathways in both polypoid-type and flat-type early-stage colorectal cancers. Journal of experimental & clinical cancer research : CR. 2006;25(2):235-42.

17. Ching-Shian Leong V, Jabal MF, Leong PP, Abdullah MA, Gul YA, Seow HF. PIK3CA gene mutations in breast carcinoma in Malaysian patients. Cancer genetics and cytogenetics. 2008;187(2):74-9.

18. Seth R, Crook S, Ibrahem S, Fadhil W, Jackson D, Ilyas M. Concomitant mutations and splice variants in KRAS and BRAF demonstrate complex perturbation of the Ras/Raf signalling pathway in advanced colorectal cancer. Gut. 2009;58(9):1234-41.

19. Kim MS, Kim SS, Ahn CH, Yoo NJ, Lee SH. Frameshift mutations of Wnt pathway genes AXIN2 and TCF7L2 in gastric carcinomas with high microsatellite instability. Human Pathology. 2009;40(1):58-64.

20. Packham D, Ward RL, Ap Lin V, Hawkins NJ, Hitchins MP. Implementation of novel pyrosequencing assays to screen for common mutations of BRAF and KRAS in a cohort of sporadic colorectal cancers. Diagnostic Molecular Pathology. 2009;18(2):62-71.

21. Baldus SE, Schaefer KL, Engers R, Hartleb D, Stoecklein NH, Gabbert HE. Prevalence and heterogeneity of KRAS, BRAF, and PIK3CA mutations in primary colorectal adenocarcinomas and their corresponding metastases. Clinical Cancer Research. 2010;16(3):790-9.

22. Irahara N, Baba Y, Nosho K, Shima K, Yan L, Dias-Santagata D, et al. NRAS mutations are rare in colorectal cancer. Diagnostic Molecular Pathology. 2010;19(3):157-63.

23. Smith G, Bounds R, Wolf H, Steele RJC, Carey FA, Wolf CR. Activating K-Ras mutations outwith hotspot codons in sporadic colorectal tumours-implications for personalised cancer medicine. British Journal of Cancer. 2010;102(4):693-703.

24. Liao W, Liao Y, Zhou JX, Xie J, Chen J, Huang W, et al. Gene mutations in epidermal growth factor receptor signaling network and their association with survival in Chinese patients with metastatic colorectal cancers. Anatomical record (Hoboken, NJ : 2007). 2010;293(9):1506-11.

25. Sameer AS, Chowdri NA, Syeed N, Banday MZ, Shah ZA, Siddiqi MA. SMAD4--molecular gladiator of the TGF-beta signaling is trampled upon by mutational insufficiency in colorectal carcinoma of Kashmiri population: an analysis with relation to KRAS proto-oncogene. BMC cancer. 2010;10:300.

26. Watanabe T, Kobunai T, Yamamoto Y, Matsuda K, Ishihara S, Nozawa K, et al. Differential gene expression signatures between colorectal cancers with and without KRAS mutations: crosstalk between the KRAS pathway and other signalling pathways. European journal of cancer (Oxford, England : 1990). 2011;47(13):1946-54.

27. Metzger B, Chambeau L, Begon DY, Faber C, Kayser J, Berchem G, et al. The human epidermal growth factor receptor (EGFR) gene in European patients with advanced colorectal cancer harbors infrequent mutations in its tyrosine kinase domain. BMC Medical Genetics. 2011;12.

28. Naghibalhossaini F, Hosseini HM, Mokarram P, Zamani M. High frequency of genes' promoter methylation, but lack Of BRAF V600E Mutation among Iranian colorectal cancer patients. Pathology and Oncology Research. 2011;17(4):819-25.

29. Khiari M, Arfaoui A, Kriaa L, Chaar I, Amara S, Lounis MA, et al. The prognostic value of the immunohistochemical expression and mutational pattern of the key mediator of Wnt signaling: beta-catenin in Tunisian patients with colorectal carcinoma. Applied immunohistochemistry & molecular morphology : AIMM. 2012;20(1):62-70.

30. Tai CJ, Chang CC, Jiang MC, Yeh CM, Su TC, Wu PR, et al. Clinical-pathological correlation of K-Ras mutation and ERK phosphorylation in colorectal cancer. Polish journal of pathology : official journal of the Polish Society of Pathologists. 2012;63(2):93-100.

31. Bond CE, Umapathy A, Ramsnes I, Greco SA, Zhen Zhao Z, Mallitt KA, et al. p53 mutation is common in microsatellite stable, BRAF mutant colorectal cancers. International journal of cancer. 2012;130(7):1567-76.

32. Laghi L, Bianchi P, Delconte G, Celesti G, Di Caro G, Pedroni M, et al. MSH3 protein expression and nodal status in MLH1-deficient colorectal cancers. Clinical cancer research : an official journal of the American Association for Cancer Research. 2012;18(11):3142-53.

33. Levidou G, Saetta AA, Gigelou F, Karlou M, Papanastasiou P, Stamatelli A, et al. ERK/pERK expression and B-raf mutations in colon adenocarcinomas: correlation with clinicopathological characteristics. World journal of surgical oncology. 2012;10:47.

34. Voorham QJM, Carvalho B, Spiertz AJ, Claes B, Mongera S, van Grieken NCT, et al. Comprehensive mutation analysis in colorectal flat adenomas. PLoS ONE. 2012;7(7).

35. Whitehall VLJ, Rickman C, Bond CE, Ramsnes I, Greco SA, Umapathy A, et al. Oncogenic PIK3CA mutations in colorectal cancers and polyps. International journal of cancer. 2012;131(4):813-20.

36. Gurzu S, Szentirmay Z, Toth E, Bara T, Bara T, Jr., Jung I. Serrated pathway adenocarcinomas: molecular and immunohistochemical insights into their recognition. PLoS One. 2013;8(3):e57699.

37. Fleming NI, Jorissen RN, Mouradov D, Christie M, Sakthianandeswaren A, Palmieri M, et al. SMAD2, SMAD3 and SMAD4 mutations in colorectal cancer. Cancer Research. 2013;73(2):725-35.

38. Aissi S, Buisine MP, Zerimech F, Kourda N, Moussa A, Manai M, et al. KRAS mutations in colorectal cancer from Tunisia: Relationships with clinicopathologic variables and data on P53 mutations and microsatellite instability. Molecular Biology Reports. 2013;40(11):6107-12.

39. Han SW, Kim HP, Shin JY, Jeong EG, Lee WC, Lee KH, et al. Targeted sequencing of cancer-related genes in colorectal cancer using next-generation sequencing. PLoS One. 2013;8(5):e64271.

40. Neumann J, Wehweck L, Maatz S, Engel J, Kirchner T, Jung A. Alterations in the EGFR pathway coincide in colorectal cancer and impact on prognosis. Virchows Archiv : an international journal of pathology. 2013;463(4):509-23.

41. Shen Y, Wang J, Han X, Yang H, Wang S, Lin D, et al. Effectors of epidermal growth factor receptor pathway: the genetic profiling ofKRAS, BRAF, PIK3CA, NRAS mutations in colorectal cancer characteristics and personalized medicine. PLoS One. 2013;8(12):e81628.

42. Yip WK, Choo CW, Leong VC, Leong PP, Jabar MF, Seow HF. Molecular alterations of Ras-Raf-mitogen-activated protein kinase and phosphatidylinositol 3-kinase-Akt signaling pathways in colorectal cancers from a tertiary hospital at Kuala Lumpur, Malaysia. APMIS : acta pathologica, microbiologica, et immunologica Scandinavica. 2013;121(10):954-66.

43. Chen TH, Chang SW, Huang CC, Wang KL, Yeh KT, Liu CN, et al. The prognostic significance of APC gene mutation and miR-21 expression in advanced-stage colorectal cancer. Colorectal disease : the official journal of the Association of Coloproctology of Great Britain and Ireland. 2013;15(11):1367-74.

44. Saigusa S, Inoue Y, Tanaka K, Toiyama Y, Okugawa Y, Shimura T, et al. Decreased expression of DUSP4 is associated with liver and lung metastases in colorectal cancer. Medical oncology (Northwood, London, England). 2013;30(3):620.

45. Shi Y, Li J, Wu SY, Qin L, Jiao YF. BRAF mutation is associated with the unique morphology of traditional serrated adenoma of the colorectum. International journal of surgical pathology. 2013;21(5):442-8.

46. Zhu K, Yan H, Wang R, Zhu H, Meng X, Xu X, et al. Mutations of KRAS and PIK3CA as independent predictors of distant metastases in colorectal cancer. Medical Oncology. 2014;31(7).

47. Chang LC, Chiu HM, Shun CT, Liang JT, Lin JT, Chen CC, et al. Mutational profiles of different macroscopic subtypes of colorectal adenoma reveal distinct pathogenetic roles for KRAS, BRAF and PIK3CA. BMC Gastroenterology. 2014;14(1).

48. Ahn TS, Jeong D, Son MW, Jung H, Park S, Kim H, et al. The BRAF mutation is associated with the prognosis in colorectal cancer. Journal of Cancer Research and Clinical Oncology. 2014;140(11):1863-71.

49. Tong JH, Lung RW, Sin FM, Law PP, Kang W, Chan AW, et al. Characterization of rare transforming KRAS mutations in sporadic colorectal cancer. Cancer biology & therapy. 2014;15(6):768-76.

50. Asl JM, Almasi S, Tabatabaiefar MA. High frequency of BRAF proto-oncogene hot spot mutation V600E in cohort of colorectal cancer patients from Ahvaz City, southwest Iran. Pakistan journal of biological sciences : PJBS. 2014;17(4):565-9.

51. Chen J, Guo F, Shi X, Zhang L, Zhang A, Jin H, et al. BRAF V600E mutation and KRAS codon 13 mutations predict poor survival in Chinese colorectal cancer patients. BMC cancer. 2014;14:802.

52. Lee SY, Haq F, Kim D, Jun C, Jo HJ, Ahn SM, et al. Comparative genomic analysis of primary and synchronous metastatic colorectal cancers. PLoS One. 2014;9(3):e90459.

53. Kawamata H, Yamashita K, Kojo K, Ushiku H, Ooki A, Watanabe M. Discrepancies between the K-ras mutational status of primary colorectal cancers and corresponding liver metastases are found in codon 13. Genomics. 2015;106(2):71-5.

54. Lan YT, Jen-Kou L, Lin CH, Yang SH, Lin CC, Wang HS, et al. Mutations in the RAS and PI3K pathways are associated with metastatic location in colorectal cancers. Journal of Surgical Oncology. 2015;111(7):905-10.

55. Samara M, Kapatou K, Ioannou M, Kostopoulou Ε, Papamichali R, Papandreou C, et al. Mutation profile of KRAS and BRAF genes in patients with colorectal cancer: Association with morphological and prognostic criteria. Genetics and Molecular Research. 2015;14(4):16793-802.

56. Abdelmaksoud-Damak R, Miladi-Abdennadher I, Triki M, Khabir A, Charfi S, Ayadi L, et al. Expression and mutation pattern of beta-catenin and adenomatous polyposis coli in colorectal cancer patients. Archives of medical research. 2015;46(1):54-62.

57. Kawazoe A, Shitara K, Fukuoka S, Kuboki Y, Bando H, Okamoto W, et al. A retrospective observational study of clinicopathological features of KRAS, NRAS, BRAF and PIK3CA mutations in Japanese patients with metastatic colorectal cancer. BMC cancer. 2015;15:258.

58. Lin EI, Tseng LH, Gocke CD, Reil S, Le DT, Azad NS, et al. Mutational profiling of colorectal cancers with microsatellite instability. Oncotarget. 2015;6(39):42334-44.

59. Grellety T, Gros A, Pedeutour F, Merlio JP, Duranton-Tanneur V, Italiano A, et al. Challenging a dogma: co-mutations exist in MAPK pathway genes in colorectal cancer. Virchows Archiv : an international journal of pathology. 2016;469(4):459-64.

60. Dallol A, Buhmeida A, Al-Ahwal MS, Al-Maghrabi J, Bajouh O, Al-Khayyat S, et al. Clinical significance of frequent somatic mutations detected by high-throughput targeted sequencing in archived colorectal cancer samples. Journal of Translational Medicine. 2016;14(1).

61. Jauhri M, Bhatnagar A, Gupta S, Shokeen Y, Minhas S, Aggarwal S. Targeted molecular profiling of rare genetic alterations in colorectal cancer using next-generation sequencing. Medical oncology (Northwood, London, England). 2016;33(10):106.

62. Nam SK, Yun S, Koh J, Kwak Y, Seo AN, Park KU, et al. BRAF, PIK3CA, and HER2 Oncogenic Alterations According to KRAS Mutation Status in Advanced Colorectal Cancers with Distant Metastasis. PLoS One. 2016;11(3):e0151865.

63. Ziv E, Bergen M, Yarmohammadi H, Boas FE, Petre EN, Sofocleous CT, et al. PI3K pathway mutations are associated with longer time to local progression after radioembolization of colorectal liver metastases. Oncotarget. 2017;8(14):23529-38.

64. Mizuno T, Cloyd JM, Vicente D, Omichi K, Chun YS, Kopetz SE, et al. SMAD4 gene mutation predicts poor prognosis in patients undergoing resection for colorectal liver metastases. European Journal of Surgical Oncology. 2018;44(5):684-92.

65. Yang Q, Huo S, Sui Y, Du Z, Zhao H, Liu Y, et al. Mutation status and immunohistochemical correlation of KRAS, NRAS, and BRAF in 260 Chinese colorectal and gastric cancers. Frontiers in Oncology. 2018;8(OCT).
