# Supplementary material for: Signal transduction pathway mutations in gastrointestinal (GI) cancers: a systematic review and meta-analysis
Source: Sci Rep. 2020 Oct 30;10:18713. doi: 10.1038/s41598-020-73770-1 (PMC7599243; doi:10.1038/s41598-020-73770-1)
Supplement: Supplementary file 4 — Supplementary Table 4. [file 41598_2020_73770_MOESM4_ESM.docx]

**Supplementary table 4. Liver malignancy (LM) signaling pathway mutation studies analysis (n=21)**

| **No** | **First**  **Author’s** | **Year** | **Country** | **Population** | | | | **Mutation Analysis** | | | | **Clinic-pathological assocoation with mutations** | **survival rate** | **Method of detection** | **Ref.** |
| --- | --- | --- | --- | --- | --- | --- | --- | --- | --- | --- | --- | --- | --- | --- | --- |
|  |  |  |  | **Sample**  **Size** | **Mean Age (y) ± SD (Range)** | **Male**  **N (%)** | **Female**  **N (%)** | **Pathway** | **Gene name** | **Exon name** | **Mutation Positive Population %** |  |  |  |  |
| 1 | Kondo | 1999 | Japan | 38 | - | - | - | Wnt | beta-catenin | 3 | 39 | beta-catenin mutation associated with portal vein involvement by tumor | - | PCR-SSCP, PCR-SS | (1) |
| 2 | Huang | 1999 | France | 22 HCC  associated with HCV | 62.7±6.3 (49 –75) | 17(77.3) | 5(22.7) | Wnt | beta-catenin | 3 | 41 | - | - | PCR-SSCP, PCR-SS | (2) |
|  |  |  |  |  |  |  |  |  | APC | codon 1493 | 0 |  |  |  |  |
| 3 | Wong | 2001 | China | 60 | 54 (28 –74) | 46 | 14 | Wnt | beta-catenin | 3 | 12 | **-** | **-** | PCR-SS | (3) |
| 4 | Kawate | 2001 | Japan | 52 | - | - | - | Smad (TGF-β) | SMAD6, 7 | 1-4 | 0 | - | - | PCR-SSCP, PCR-SS | (4) |
| 5 | Chen | 2002 | Taiwan | 10 | - | - | - | Wnt | beta-catenin | 3,4 | 30 | - | - | PCR-SS | (5) |
| 6 | Taniguchi | 2002 | USA | 73 HCC | - | 42 | 31 | Wnt | beta-catenin | - | 19.2 | - | - | PCR-SS, HPLC | (6) |
|  |  |  |  |  |  |  |  |  | AXIN1 |  | 9.6 |  |  |  |  |
|  |  |  |  |  |  |  |  |  | AXIN2 |  | 2.7 |  |  |  |  |
|  |  |  |  | 28 hepatoblastoma |  |  |  |  | AXIN1 |  | 7.4 |  |  |  |  |
|  |  |  |  |  |  |  |  |  | beta-catenin |  | 70.4 |  |  |  |  |
|  |  |  |  |  |  |  |  |  | AXIN2 |  | - |  |  |  |  |
|  |  |  |  |  |  |  |  |  | CTNNB1 | 3 | 70 |  |  |  |  |
| 7 | Tannapfel | 2003 | Germany | 25 HCC | 69 | - | - | MAPK | KRAS | 2-18 | 0 | - | Correlation not identified | PCR-SS | (7) |
|  |  |  |  |  |  |  |  |  | BRAF |  |  |  |  |  |  |
| 8 | Kim | 2008 | Korea | 36 | 57.7 ( 34–71) | 32 | 4 | Wnt | AXIN1 | 3-5 | 25 | - | - | PCR-SS, IHC | (8) |
|  |  |  |  |  |  |  |  |  | beta-catenin | 3 | 2.8 |  |  |  |  |
| 9 | Xie | 2009 | Korea | 48 | - | - | - | JAK/STAT | JAK1 | All 24 exons | 1.2 | - | - | PCR-SSCP | (9) |
| 10 | Cieply | 2009 | USA | 32 | - | - | - | Wnt | CTNNB1 | 3 | 28 | CTNNB1 mutations are associated with vascular involvement and tumor size | - | PCR-SS | (10) |
| 11 | Purcell | 2011 | Australia | 84 hepatoblastoma | - | - | - | Wnt | CTNNB1 | 3 | 15 | - | - | PCR-SS | (11) |
| 12 | Ueda | 2011 | Japan | 56 hepatoblastoma | median 1.3 | 36 | 20 | Wnt | CTNNB1 | 3 | 75 | - | - | PCR-SS | (12) |
| 13 | Li | 2012 | China | 90 | - | 74 | 16 | PI3 | PIK3CA | 9 | 1.1 | - | - | PCR-SS | (13) |
|  |  |  |  |  |  |  |  |  |  | 20 | 0 |  |  |  |  |
| 14 | Kan | 2013 | USA | 88 HCC, 81 of them had HBV | - | - | - | Wnt and JAK/STAT | beta-catenin | - | 15.9 | - | - | WGS | (14) |
|  |  |  |  |  |  |  |  |  | JAK1 |  | 9.1 |  |  |  |  |
|  |  |  |  |  |  |  |  | p53 signaling | p53 |  | 35.2 |  |  |  |  |
| 15 | Long | 2013 | China | 116 HCV associated HCC | 52.8±9.1 | - | - | p53 signaling | p53 | 5-8 | 16.4 | p53 mutations were associated with negative HCV result | - | PCR-SSCP, PCR-SS | (15) |
| 16 | Jia | 2014 | China | 6 Hepatoblastoma | 19 | 3 | 3 | Wnt | CTNNB1 |  | 23-31 | - | - | NGS | (16) |
|  |  |  |  |  |  |  |  |  | CAPRIN2 |  | 19 |  |  |  |  |
|  |  |  |  |  |  |  |  |  | NYAP2 |  | 22 |  |  |  |  |
|  |  |  |  |  |  |  |  |  | RNF169 |  | 12 |  |  |  |  |
|  |  |  |  |  |  |  |  |  | GLTSCR1 |  | 41 |  |  |  |  |
|  |  |  |  |  |  |  |  |  | KLHL22 |  | 35.9 |  |  |  |  |
|  |  |  |  |  |  |  |  |  | VCAM1 |  | 34 |  |  |  |  |
|  |  |  |  |  |  |  |  |  | HDAC9 |  | 32 |  |  |  |  |
|  |  |  |  |  |  |  |  |  | FGGY |  | 32.7 |  |  |  |  |
|  |  |  |  |  |  |  |  |  | TRPC4AP |  | 35.4 |  |  |  |  |
| 17 | Marchio | 2014 | Peru | 80 | 42±20 | 47 | 33 | Wnt and p53 signaling and MAPK | CTNNB1 | - | 15 | p53 mutations were associated with a poor tumor differentiation/ AXIN1 mutations were associated with multinodular liver tumors/ CTNNB1 and AXIN1 associated with tumor recurrence / AXIN1 associated with gender and geographical location | Wnt pathway associated with survival | PCR-SS | (17) |
|  |  |  |  |  |  |  |  |  | AXIN1 |  | 12.5 |  |  |  |  |
|  |  |  |  |  |  |  |  |  | Wnt | - | 26 |  |  |  |  |
|  |  |  |  |  |  |  |  |  | KRAS | - | 4 |  |  |  |  |
|  |  |  |  |  |  |  |  |  | p53 | 6 | 50 |  |  |  |  |
|  |  |  |  |  |  |  |  |  | p53 | R249S | 1.2 |  |  |  |  |
|  |  |  |  |  |  |  |  |  | p53 | 4 | 61 |  |  |  |  |
|  |  |  |  |  |  |  |  |  | BRAF | - | 0 |  |  |  |  |
|  |  |  |  |  |  |  |  |  | HRAS | - | 3 |  |  |  |  |
|  |  |  |  |  |  |  |  |  | NRAS | - | 0 |  |  |  |  |
|  |  |  |  |  |  |  |  |  | NFE2L2 | - | 1.2 |  |  |  |  |
|  |  |  |  |  |  |  |  |  | ARID2 | - | 1.2 |  |  |  |  |
| 18 | Lu | 2015 | China | 12 | 51(33-65) | 11 | 1 | Wnt and P53 signaling and MAPK | KRAS | - | 16.6 | - | - | PCR-SS | (18) |
|  |  |  |  |  |  |  |  |  | JAK |  | 16.6 |  |  |  |  |
|  |  |  |  |  |  |  |  |  | RUNX1 |  | 25 |  |  |  |  |
|  |  |  |  |  |  |  |  |  | NOTCH1 |  | 16.6 |  |  |  |  |
|  |  |  |  |  |  |  |  |  | p53 |  | 41 |  |  |  |  |
| 19 | Suarez | 2015 | France | 54 | 65 ± 8 | - | - | Wnt | beta-catenin | - | 18.8 | - | - | PCR-SS | (19) |
| 20 | Okabe | 2016 | USA | 125 | - | - | - | Wnt | CTNNB1 | 3 | 12-12.8 | CTNNB1 associated with Tumor stage | - | qRT-PCR, PCR-SS | (20) |
| 21 | Ho | 2017 | China | 95 | - | - | - | mTOR signaling | TSC1, TSC2 | - | 16.2 | TSC1, TSC2 associated with tumor size and invasion | - | NGS | (21) |

References:

1. Kondo Y, Kanai Y, Sakamoto M, Genda T, Mizokami M, Ueda R, et al. β‐Catenin accumulation and mutation of exon 3 of the β‐catenin gene in hepatocellular carcinoma. Japanese journal of cancer research. 1999;90(12):1301-9.

2. Huang H, Fujii H, Sankila A, Mahler-Araujo BM, Matsuda M, Cathomas G, et al. Beta-catenin mutations are frequent in human hepatocellular carcinomas associated with hepatitis C virus infection. The American journal of pathology. 1999;155(6):1795-801.

3. Wong CM, Fan ST, Ng IO. beta-Catenin mutation and overexpression in hepatocellular carcinoma: clinicopathologic and prognostic significance. Cancer. 2001;92(1):136-45.

4. Kawate S, Ohwada S, Hamada K, Koyama T, Takenoshita S, Morishita Y, et al. Mutational analysis of the Smad6 and Smad7 genes in hepatocellular carcinoma. International journal of molecular medicine. 2001;8(1):49-52.

5. Chen YW, Jeng YM, Yeh SH, Chen PJ. p53 gene and Wnt signaling in benign neoplasms: β-catenin mutations in hepatic adenoma but not in focal nodular hyperplasia. Hepatology. 2002;36(4 I):927-35.

6. Taniguchi K, Roberts LR, Aderca IN, Dong X, Qian C, Murphy LM, et al. Mutational spectrum of beta-catenin, AXIN1, and AXIN2 in hepatocellular carcinomas and hepatoblastomas. Oncogene. 2002;21(31):4863-71.

7. Tannapfel A, Sommerer F, Benicke M, Katalinic A, Uhlmann D, Witzigmann H, et al. Mutations of the BRAF gene in cholangiocarcinoma but not in hepatocellular carcinoma. Gut. 2003;52(5):706-12.

8. Kim YD, Park CH, Kim HS, Choi SK, Rew JS, Kim DY, et al. Genetic alterations of Wnt signaling pathway-associated genes in hepatocellular carcinoma. Journal of gastroenterology and hepatology. 2008;23(1):110-8.

9. Xie HJ, Bae HJ, Noh JH, Eun JW, Kim JK, Jung KH, et al. Mutational analysis of JAK1 gene in human hepatocellular carcinoma. Neoplasma. 2009;56(2):136-40.

10. Cieply B, Zeng G, Proverbs-Singh T, Geller DA, Monga SPS. Unique phenotype of hepatocellular cancers with exon-3 mutations in beta-catenin gene. Hepatology. 2009;49(3):821-31.

11. Purcell R, Childs M, Maibach R, Miles C, Turner C, Zimmermann A, et al. HGF/c-Met related activation of beta-catenin in hepatoblastoma. Journal of experimental & clinical cancer research : CR. 2011;30:96.

12. Ueda Y, Hiyama E, Kamimatsuse A, Kamei N, Ogura K, Sueda T. Wnt signaling and telomerase activation of hepatoblastoma: correlation with chemosensitivity and surgical resectability. Journal of pediatric surgery. 2011;46(12):2221-7.

13. Li X, Zhang Q, He W, Meng W, Yan J, Zhang L, et al. Low frequency of PIK3CA gene mutations in hepatocellular carcinoma in Chinese population. Pathology and Oncology Research. 2012;18(1):57-60.

14. Kan Z, Zheng H, Liu X, Li S, Barber TD, Gong Z, et al. Whole-genome sequencing identifies recurrent mutations in hepatocellular carcinoma. Genome research. 2013;23(9):1422-33.

15. Long J, Wang Y, Li M, Tong WM, Jia JD, Huang J. Correlation of P53 mutations with HCV positivity in hepatocarcinogenesis: Identification of a novel P53 microindel in hepatocellular carcinoma with HCV infection. Oncology Reports. 2013;30(1):119-24.

16. Jia D, Dong R, Jing Y, Xu D, Wang Q, Chen L, et al. Exome sequencing of hepatoblastoma reveals novel mutations and cancer genes in the Wnt pathway and ubiquitin ligase complex. Hepatology. 2014;60(5):1686-96.

17. Marchio A, Bertani S, Rojas Rojas T, Doimi F, Terris B, Deharo E, et al. A peculiar mutation spectrum emerging from young peruvian patients with hepatocellular carcinoma. PLoS One. 2014;9(12):e114912.

18. Lu J, Yin J, Dong R, Yang T, Yuan L, Zang L, et al. Targeted sequencing of cancer-associated genes in hepatocellular carcinoma using next generation sequencing. Molecular medicine reports. 2015;12(3):4678-82.

19. Suarez MI, Uribe D, Jaramillo CM, Osorio G, Perez JC, Lopez R, et al. Wnt/beta-catenin signaling pathway in hepatocellular carcinomas cases from Colombia. Annals of hepatology. 2015;14(1):64-74.

20. Okabe H, Kinoshita H, Imai K, Nakagawa S, Higashi T, Arima K, et al. Diverse Basis of beta-Catenin Activation in Human Hepatocellular Carcinoma: Implications in Biology and Prognosis. PLoS One. 2016;11(4):e0152695.

21. Ho DWH, Chan LK, Chiu YT, Xu IMJ, Poon RTP, Cheung TT, et al. TSC1/2 mutations define a molecular subset of HCC with aggressive behaviour and treatment implication. Gut. 2017;66(8):1496-506.
