# Supplementary material for: Signal transduction pathway mutations in gastrointestinal (GI) cancers: a systematic review and meta-analysis
Source: Sci Rep. 2020 Oct 30;10:18713. doi: 10.1038/s41598-020-73770-1 (PMC7599243; doi:10.1038/s41598-020-73770-1)
Supplement: Supplementary file 5 — Supplementary Table 5. [file 41598_2020_73770_MOESM5_ESM.docx]

**Supplementary table 5. Pancreatic cancer (PC) signaling pathway mutation studies analysis (n=9)**

| **No.** | **First**  **Author’s** | **Year** | **Country** | **Population** | | | | **Mutation Analysis** | | | | | **Clinic-pathological** | **survival rate** | **Method of detection** | **Ref.** |
| --- | --- | --- | --- | --- | --- | --- | --- | --- | --- | --- | --- | --- | --- | --- | --- | --- |
|  |  |  |  | **Sample**  **Size** | **Mean Age (y) ± SD (Range)** | **Male**  **N (%)** | **Female N (%)** | **Pathway** | **Gene name** | **Exon name** | **Mutation Positive Population %** | |  |  |  |  |
| 1 | Abraham | 2002 | USA | 21 | 59 | 15 | 6 | Wnt | APC/beta-catenin | 3 | 23.5 | | - | - | PCR-SS | (1) |
| 2 | Immervoll | 2005 | Norway | 43 | 66 | 25 | 18 | MAPK | KRAS | 1 | 67 | | - | Correlation was not identified | PCR-SS | (2) |
|  |  |  |  |  |  |  |  |  | BRAF | 11, 15 | 0 | |  |  |  |  |
|  |  |  |  |  |  |  |  |  | EGRF | 18-21 | 0 | |  |  |  |  |
| 3 | Nomoto | 2008 | Japan | 32 | - | 18 | 14 | Smad (TGF-β) | RUNX3 | 1 | 62.5% | | Correlation was not identified | - | PCR-SSCP | (3) |
| 4 | Schönleben | 2008 | USA | 38 | - | - | - | PI3, MAPK | KRAS | - | 43 | | - | Correlation was not identified | PCR-SS | (4) |
|  |  |  |  |  |  |  |  |  | BRAF |  | 2.7 | |  |  |  |  |
|  |  |  |  |  |  |  |  |  | PIK3CA |  | 11 | |  |  |  |  |
| 5 | Schonleben | 2008 | Germany | 36  IPMN/IPMNC | 68.1( 41–84) | 24 | 14 | PI3, MAPK | PIK3CA | 1, 4, 5, 6, 7, 9, 12, 18, 20 | 11 | | - | - | PCR-SS | (5) |
|  |  |  |  |  |  |  |  |  | KRAS | 1 | 47 | |  |  |  |  |
|  |  |  |  |  |  |  |  |  | BRAF | 5, 11, 15 | 2.7 | |  |  |  |  |
| 6 | Mohri | 2012 | Japan | 25 PMN | 66 ± 6.8 | 9 | 2 | MAPK | KRAS | 2 | intestinal | 27 | KRAS mutations were associated with gastric type IPMN | - | PCR-SS | (6) |
|  |  |  |  |  |  |  |  |  |  |  | gastric | 81 |  |  |  |  |
| 7 | Garcia-Carracedo | 2013 | USA | 36 | 70 (44 to 85) | 20 | 16 | PI3 | PIK3CA | 9, 20 | 2.7 | | - | - | PCR-SS | (7) |
|  |  |  |  |  |  |  |  |  | AKT1 | exons 2 | 8.3 | |  |  |  |  |
| 8 | Mikhitarian | 2014 | USA | 52 ampullary adenocarcinomas | 52-72 | 33 | 19 | MAPK and Smad | KRAS | - | 42 | | - | - | Capillary electrophoresis | (8) |
|  |  |  |  |  |  |  |  |  | BRAF | - | 0 | |  |  |  |  |
|  |  |  |  |  |  |  |  |  | PIK3CA | 9 | 12 | |  |  |  |  |
|  |  |  |  |  |  |  |  |  | SMAD4 | - | 6 | |  |  |  |  |
| 9 | Witkiewicz | 2015 | USA | 109 ductal adenocarcinoma | - | - | - | PI3, MAPK, Wnt | KRAS | - | 92 | | - | KRAS mutations were associated with poor outcome | WES | (9) |
|  |  |  |  |  |  |  |  |  | BCLAF1 |  | 5 | |  |  |  |  |
|  |  |  |  |  |  |  |  |  | IRF6 |  | 4 | |  |  |  |  |
|  |  |  |  |  |  |  |  |  | FLG |  | 10 | |  |  |  |  |
|  |  |  |  |  |  |  |  |  | AXIN1 |  | 5 | |  |  |  |  |
|  |  |  |  |  |  |  |  |  | GLI3 |  | 6 | |  |  |  |  |
|  |  |  |  |  |  |  |  |  | PIK3CA |  | 4 | |  |  |  |  |

References:

1. Abraham SC, Wu TT, Hruban RH, Lee JH, Yeo CJ, Conlon K, et al. Genetic and immunohistochemical analysis of pancreatic acinar cell carcinoma: frequent allelic loss on chromosome 11p and alterations in the APC/beta-catenin pathway. The American journal of pathology. 2002;160(3):953-62.

2. Immervoll H, Hoem D, Kugarajh K, Steine SJ, Molven A. Molecular analysis of the EGFR-RAS-RAF pathway in pancreatic ductal adenocarcinomas: Lack of mutations in the BRAF and EGFR genes. Virchows Archiv. 2006;448(6):788-96.

3. Nomoto S, Kinoshita T, Mori T, Kato K, Sugimoto H, Kanazumi N, et al. Adverse prognosis of epigenetic inactivation in RUNX3 gene at 1p36 in human pancreatic cancer. Br J Cancer. 2008;98(10):1690-5.

4. Schönleben F, Allendorf JD, Qiu W, Li X, Ho DJ, Ciau NT, et al. Mutational analyses of multiple oncogenic pathways in intraductal papillary mucinous neoplasms of the pancreas. Pancreas. 2008;36(2):168-72.

5. Schonleben F, Qiu W, Remotti HE, Hohenberger W, Su GH. PIK3CA, KRAS, and BRAF mutations in intraductal papillary mucinous neoplasm/carcinoma (IPMN/C) of the pancreas. Langenbeck's archives of surgery. 2008;393(3):289-96.

6. Mohri D, Asaoka Y, Ijichi H, Miyabayashi K, Kudo Y, Seto M, et al. Different subtypes of intraductal papillary mucinous neoplasm in the pancreas have distinct pathways to pancreatic cancer progression. Journal of gastroenterology. 2012;47(2):203-13.

7. Garcia-Carracedo D, Turk AT, Fine SA, Akhavan N, Tweel BC, Parsons R, et al. Loss of PTEN expression is associated with poor prognosis in patients with intraductal papillary mucinous neoplasms of the pancreas. Clinical cancer research : an official journal of the American Association for Cancer Research. 2013;19(24):6830-41.

8. Mikhitarian K, Pollen M, Zhao Z, Shyr Y, Merchant NB, Parikh A, et al. Epidermal growth factor receptor signaling pathway is frequently altered in ampullary carcinoma at protein and genetic levels. Modern pathology : an official journal of the United States and Canadian Academy of Pathology, Inc. 2014;27(5):665-74.

9. Witkiewicz AK, McMillan EA, Balaji U, Baek G, Lin WC, Mansour J, et al. Whole-exome sequencing of pancreatic cancer defines genetic diversity and therapeutic targets. Nature Communications. 2015;6.
