# Supplementary material for: Signal transduction pathway mutations in gastrointestinal (GI) cancers: a systematic review and meta-analysis
Source: Sci Rep. 2020 Oct 30;10:18713. doi: 10.1038/s41598-020-73770-1 (PMC7599243; doi:10.1038/s41598-020-73770-1)
Supplement: Supplementary file 6 — Supplementary Table 6. [file 41598_2020_73770_MOESM6_ESM.docx]

**Supplementary table 6. Other GI cancer signaling pathway mutation studies analysis (n=15)**

| **No** | **Location** | **GI cancer type** | **First**  **Author** | **Year** | **Country** | **Population** | | | | **Mutation Analysis** | | | | **Clinic-pathological** | **survival rate** | **Method of detection** | **Ref.** |
| --- | --- | --- | --- | --- | --- | --- | --- | --- | --- | --- | --- | --- | --- | --- | --- | --- | --- |
|  |  |  |  |  |  | **Sample size** | **Mean Age ± SD**  **(Range)** | **Male N (%)** | **Female**  **N (%)** | **Pathway** | **Gene name** | **Exon** | **Mutation Positive Population %** |  |  |  |  |
| 1 | Extra GI | biliary tract | Rashid | 2001 | China | 107 | 64.6±8.9 | 64 | 43 | Wnt | beta-catenin | - | 7.5 | beta-catenin mutations were associated with tumor location and stage | beta-catenin mutations were associated with better outcome | PCR-SS | (1) |
| 2 | Extra GI | Cholangiocarcinoma | Tannapfel | 2003 | Germany | 69 | 69 | - | - | MAPK | KRAS | 2-18 | 22 | - | Correlation was not identified | PCR-SS | (2) |
|  |  |  |  |  |  |  |  |  |  |  | BRAF |  | 45 |  |  |  |  |
| 3 | GI | small intestinal adenocarcinomas | Bläker | 2004 | Germany | 21 | - | - | - | Wnt, MAPK | APC |  | 9.5 | - | - | PCR-SS | (3) |
|  |  |  |  |  |  |  |  |  |  |  | CTNNB |  | 4.7 |  |  |  |  |
|  |  |  |  |  |  |  |  |  |  |  | KRAS |  | 57 |  |  |  |  |
|  |  |  |  |  |  |  |  |  |  |  | BRAF |  | 4.7 |  |  |  |  |
| 4 | Extra GI | Salivary Gland  Carcinomas | Dahse | 2009 | Germany | 65 | - | - | - | MAPK | KRAS | - | 1.5 | - | - | PCR-SS | (4) |
|  |  |  |  |  |  |  |  |  |  |  | BRAF |  | 0 |  |  |  |  |
| 5 | GI | gastroesophageal cancer | Catenacci | 2011 | USA | gastroesophageal adenocarcinoma 94 | - | - | - | RON | MST1R | - | 11 | - | - | PCR-SS, IHC, CISH | (5) |
| 6 | GI | squamous cell anal carcinoma | Paliga | 2012 | Canada | 90 | 59.5 (37–88) | 33 | 57 | MAPK | KRAS | - | 0 | - | - | PCR-SS | (6) |
|  |  |  |  |  |  |  |  |  |  |  | EGFR |  | 0 |  |  |  |  |
| 7 | GI | rectal cancer | Ree | 2012 | Norway | 79 rectal cancer | - | - | - | MAPK | KRAS | 2 | 35 | - | - | PCR-SS | (7) |
|  |  |  |  |  |  |  |  |  |  |  | BRAF | 15 | 6.3 |  |  |  |  |
|  |  |  |  |  |  |  |  |  |  |  | PIK3CA | 9, 20 | 9.5 |  |  |  |  |
|  |  |  |  |  |  |  |  |  |  |  | ERBB2 | - | 3.2 |  |  |  |  |
| 8 | Extra GI | ICC | Gao | 2014 | China | 124 | - | - | - | PTP | PTPN3 | - | 41.1 | - | - | WES | (8) |
|  |  |  |  |  |  |  |  |  |  | PTP | RS, RB, RQ, z1, 3 |  | 51.6 |  |  |  |  |
|  |  |  |  |  |  |  |  |  |  | P53 signaling | p53 | - | 31.5 |  |  |  |  |
|  |  |  |  |  |  |  |  |  |  | MAPK | KRAS | - | 7.3 |  |  |  |  |
| 9 | GI | ESCC | Gao | 2014 | China | 113 | - | - | - | Hippo Signaling | FAT1, 2, 3, 4, p53, CCND1, CDKN2, NFE2L2, RB1 | - | 27 | - | - | WES | (8) |
|  |  |  |  |  |  |  |  |  |  |  | AJUBA |  | 7 |  |  |  |  |
|  |  |  |  |  |  |  |  |  |  | NOTCH signaling | NOTCH1,2,3 |  | 22 |  |  |  |  |
|  |  |  |  |  |  |  |  |  |  |  | FBXW7 |  | 5 |  |  |  |  |
| 10 | Extra GI | GBC | Li | 2014 | China | 57 | - | - | - | MAPK | KRAS | - | 7.8 | - | - | WES | (9) |
|  |  |  |  |  |  |  |  |  |  |  | ERBB3 |  | 11.8 |  |  |  |  |
|  |  |  |  |  |  |  |  |  |  | P53 signaling | p53 |  | 47 |  |  |  |  |
| 11 | GI | esophagus | Saito | 2014 | Japan | 30 Basaloid squamous cell carcinoma | - | - | - | Wnt | APC | - | 10 | - | - | PCR-SS | (10) |
|  |  |  |  |  |  |  |  |  |  |  | CTNNB1 | 3 | 0 |  |  |  |  |
|  |  |  |  |  |  |  |  |  |  |  | AXIN1 | - | 6.7 |  |  |  |  |
|  |  |  |  |  |  |  |  |  |  |  | AXIN2 | - | 6.7 |  |  |  |  |
| 12 | Extra GI | Intraductal papillary neoplasm of the bile duct | Schlitter | 2014 | Germany. | 45 | 64 | - | - | Wnt and MAPK | KRAS | 2,3 | 29 | - | - | real-time PCR, PCR-SS | (11) |
|  |  |  |  |  |  |  |  |  |  |  | BRAF | - | 0 |  |  |  |  |
|  |  |  |  |  |  |  |  |  |  |  | beta-catenin | - | 7.5 |  |  |  |  |
|  |  |  |  |  |  |  |  |  |  |  | SMAD4 | - | 7.5 |  |  |  |  |
|  |  | Cholangiocarcinoma |  |  |  | 22 | 67 |  |  |  | KRAS | 2,3 | 14 |  |  |  |  |
|  |  |  |  |  |  |  |  |  |  |  | BRAF | - | 0 |  |  |  |  |
|  |  |  |  |  |  |  |  |  |  |  | beta-catenin | - | 0 |  |  |  |  |
|  |  |  |  |  |  |  |  |  |  |  | SMAD4 | - | 14 |  |  |  |  |
| 13 | GI | duodenal adenocarcinoma | Yuan | 2016 | China | 12 | 56.8 | 7 | 5 | Wnt, MAPK, P53 signaling | p53 |  | 58.3 | - | - | NGS | (12) |
|  |  |  |  |  |  |  |  |  |  |  | KRAS |  | 25 |  |  |  |  |
|  |  |  |  |  |  |  |  |  |  |  | ARID2 |  | 25 |  |  |  |  |
|  |  |  |  |  |  |  |  |  |  |  | ADAMTS17 |  | 16.7 |  |  |  |  |
|  |  |  |  |  |  |  |  |  |  |  | CDHR1 |  | 25 |  |  |  |  |
|  |  |  |  |  |  |  |  |  |  |  | CTNNB1 |  | 25 |  |  |  |  |
|  |  |  |  |  |  |  |  |  |  |  | APC |  | 16.7 |  |  |  |  |
|  |  |  |  |  |  |  |  |  |  |  | BOK |  | 16.7 |  |  |  |  |
|  |  |  |  |  |  |  |  |  |  |  | RTDR1 |  | 16.7 |  |  |  |  |
|  |  |  |  |  |  |  |  |  |  |  | BTN3A2 |  | 16.7 |  |  |  |  |
| 14 | GI | small bowel adenocarcinoma MSS | Hänninen | 2018 | Finland | 106 | 62 (24- 86) | 53 | 53 | Wnt, MAPK, P53 signaling | p53 |  | 48 | - | - | NGS | (13) |
|  |  |  |  |  |  |  |  |  |  |  | KRAS |  | 47 |  |  |  |  |
|  |  |  |  |  |  |  |  |  |  |  | APC |  | 22 |  |  |  |  |
|  |  |  |  |  |  |  |  |  |  |  | SMAD4 |  | 15 |  |  |  |  |
|  |  |  |  |  |  |  |  |  |  |  | SOX9 |  | 12 |  |  |  |  |
|  |  |  |  |  |  |  |  |  |  |  | BRAF |  | 11 |  |  |  |  |
|  |  |  |  |  |  |  |  |  |  |  | ERBB2 |  | 11 |  |  |  |  |
|  |  | small bowel adenocarcinoma MSI |  |  |  |  |  |  |  |  | ACVR2A |  | 87 |  |  |  |  |
|  |  |  |  |  |  |  |  |  |  |  | BMPR2 |  | 60 |  |  |  |  |
|  |  |  |  |  |  |  |  |  |  |  | KRAS |  | 53 |  |  |  |  |
|  |  |  |  |  |  |  |  |  |  |  | APC |  | 47 |  |  |  |  |
| 15 | GI | rectal cancer | Yang | 2018 | China | 140 | 61.5±10.39 | 99 | 41 | MAPK | KRAS | 2 | 37.1 | KRAS mutations were associated with gender, tumor location and tumor differentiation |  | ARMS-PCR | (14) |
|  |  |  |  |  |  |  |  |  |  |  | NRAS | 2,3,4 | 4.3 |  |  |  |  |
|  |  |  |  |  |  |  |  |  |  |  | BRAF | 15 | 0.7 |  |  |  |  |

References:

1. Rashid A, Gao YT, Bhakta S, Shen MC, Wang BS, Deng J, et al. β-Catenin mutations in biliary tract cancers: A population-based study in China. Cancer Research. 2001;61(8):3406-9.

2. Tannapfel A, Sommerer F, Benicke M, Katalinic A, Uhlmann D, Witzigmann H, et al. Mutations of the BRAF gene in cholangiocarcinoma but not in hepatocellular carcinoma. Gut. 2003;52(5):706-12.

3. Bläker H, Helmchen B, Bönisch A, Aulmann S, Penzel R, Otto HF, et al. Mutational activation of the RAS-RAF-MAPK and the wnt pathway in small intestinal adenocarcinomas. Scandinavian Journal of Gastroenterology. 2004;39(8):748-53.

4. Dahse R, Kromeyer-Hauschild K, Berndt A, Kosmehl H. No incidence of BRAF mutations in salivary gland carcinomasImplications for anti-EGFR Therapies. Journal of Biomedicine and Biotechnology. 2009;2009.

5. Catenacci DV, Cervantes G, Yala S, Nelson EA, El-Hashani E, Kanteti R, et al. RON (MST1R) is a novel prognostic marker and therapeutic target for gastroesophageal adenocarcinoma. Cancer biology & therapy. 2011;12(1):9-46.

6. Paliga A, Onerheim R, Gologan A, Chong G, Spatz A, Niazi T, et al. EGFR and K-ras gene mutation status in squamous cell anal carcinoma: A role for concurrent radiation and EGFR inhibitors. British Journal of Cancer. 2012;107(11):1864-8.

7. Ree AH, Kristensen AT, Saelen MG, de Wijn R, Edvardsen H, Jovanovic J, et al. Tumor phosphatidylinositol-3-kinase signaling and development of metastatic disease in locally advanced rectal cancer. PLoS One. 2012;7(11):e50806.

8. Gao YB, Chen ZL, Li JG, Hu XD, Shi XJ, Sun ZM, et al. Genetic landscape of esophageal squamous cell carcinoma. Nat Genet. 2014;46(10):1097-102.

9. Li M, Zhang Z, Li X, Ye J, Wu X, Tan Z, et al. Whole-exome and targeted gene sequencing of gallbladder carcinoma identifies recurrent mutations in the ErbB pathway. Nat Genet. 2014;46(8):872-6.

10. Saito T, Mitomi H, Imamhasan A, Hayashi T, Mitani K, Takahashi M, et al. Downregulation of sFRP-2 by epigenetic silencing activates the beta-catenin/Wnt signaling pathway in esophageal basaloid squamous cell carcinoma. Virchows Archiv : an international journal of pathology. 2014;464(2):135-43.

11. Schlitter AM, Born D, Bettstetter M, Specht K, Kim-Fuchs C, Riener MO, et al. Intraductal papillary neoplasms of the bile duct: stepwise progression to carcinoma involves common molecular pathways. Modern pathology : an official journal of the United States and Canadian Academy of Pathology, Inc. 2014;27(1):73-86.

12. Yuan W, Zhang Z, Dai B, Wei Q, Liu J, Liu Y, et al. Whole-exome sequencing of duodenal adenocarcinoma identifies recurrent Wnt/β-catenin signaling pathway mutations. Cancer. 2016;122(11):1689-96.

13. Hänninen UA, Katainen R, Tanskanen T, Plaketti RM, Laine R, Hamberg J, et al. Exome-wide somatic mutation characterization of small bowel adenocarcinoma. PLoS Genetics. 2018;14(3).

14. Yang Q, Huo S, Sui Y, Du Z, Zhao H, Liu Y, et al. Mutation status and immunohistochemical correlation of KRAS, NRAS, and BRAF in 260 Chinese colorectal and gastric cancers. Frontiers in Oncology. 2018;8(OCT).
